# Emotion regulation unveiled through the categorical lens of attachment

**DOI:** 10.1186/s40359-024-01748-z

**Published:** 2024-04-27

**Authors:** Marcos Domic-Siede, Mónica Guzmán-González, Andrea Sánchez-Corzo, Xaviera Álvarez, Vanessa Araya, Camila Espinoza, Karla Zenis, Jennifer Marín-Medina

**Affiliations:** 1 Laboratorio de Neurociencia Cognitiva, Escuela de Psicología, Universidad Católica del Norte, Av. Angamos 0610, Antofagasta, Chile; 2 Department of Diagnostic Imaging, St. Jude Children’s Research Hospital, Memphis, USA

**Keywords:** Attachment styles, Emotion regulation, Cognitive reappraisal, Expressive suppression, ECR-12

## Abstract

**Background:**

Emotion regulation, the process by which individuals manage and modify their emotional experiences, expressions, and responses to adaptively navigate and cope with various situations, plays a crucial role in daily life. Our study investigates the variations in emotion regulation strategies among individuals with different attachment styles (AS). Specifically, we examine how individuals with secure, anxious, avoidant, and fearful attachment styles effectively utilize cognitive reappraisal and expressive suppression to regulate their emotions.

**Methods:**

A total of *n* = 98 adults were instructed to attend, reappraise, or suppress their emotions while viewing negative and neutral images from the International Affective Picture System (IAPS) in an experimental emotion regulation task. After completing the task, participants rated the valence and arousal elicited by the images. Attachment styles were measured using the ECR-12 questionnaire and then categorized into four AS.

**Results:**

Our study revealed that individuals with secure AS (*n* = 39) effectively reduced displeasure through cognitive reappraisal but experienced levels of displeasure with expressive suppression. Anxious AS (*n* = 16) individuals successfully reduced displeasure using cognitive reappraisal but struggled to regulate arousal and effectively use expressive suppression. Avoidant AS (*n* = 24) individuals could reduce displeasure with both strategies but experienced high arousal during suppression attempts. Fearful AS (*n* = 19) individuals effectively regulated both displeasure and arousal using either strategy. However, Secure AS individuals showed superior reappraisal efficacy, significantly reducing arousal levels compared to the Fearful AS group. Both Secure and Avoidant AS groups experienced higher valence during reappraisal relative to a baseline, indicating a decrease in displeasure.

**Conclusions:**

Individuals with different AS exhibit variations in the effectiveness of their use of emotion regulation strategies. Our findings reinforce the significance of AS in shaping emotion regulation processes and emphasize the need for tailored approaches to support individuals with different attachment orientations.

**Supplementary Information:**

The online version contains supplementary material available at 10.1186/s40359-024-01748-z.

## Introduction

Over the years, researchers from various approaches and perspectives have shown interest in emotions and have defined and redefined the concept. From the perspective of cognitive theorists, it is argued that emotions arise from neocortical regions and represent a cognitive expression of bodily processes, behaviors, events, and social contexts [1]. On the other hand, according to James [2], emotions are a combination of psychological and physical aspects, as they represent involuntary and adaptive reactions shaped by experience. These emotions are triggered by external situations, objects, or stimuli and bring about changes in both the individual’s behavior and physiology. Additionally, there are researchers with constructivist approaches who suggest that emotions arise from constructions that can be psychological or social in nature [1]. According to this perspective, emotions result from internal construction processes or social influences that shape how we interpret and experience the emotional world.

From a dimensional viewpoint of emotions, Averill [3] argues that several theorists consider emotions to be conceptualized or understood through affective dimensions, which can be two or more. In this regard, the Circumplex model is presented, proposing two bipolar dimensions: valence, which indicates the degree of pleasure or displeasure associated with the emotion, and arousal, which represents the level of intensity experienced in the emotion [3].

### Emotion regulation strategies

Emotion regulation encompasses the processes employed by individuals to influence their emotional states, aiming to achieve personal goals and adapt to their environment. It involves monitoring, evaluating, and modifying emotional reactions, both internal and related to personal experience, and external, related to expression [4, 5]. These processes play a pivotal role in the initiation and modulation of emotional responses, with strategies ranging from automatic to controlled and conscious to unconscious [6].

Gross [6, 7] has proposed and categorized two main types of these strategies: antecedent-focused and response-focused. Antecedent-focused strategies, such as situation selection, situation modification, attentional deployment, and cognitive change (or cognitive reappraisal), are implemented before the full activation of emotional response. In contrast, response-focused strategies, like response modulation (or expressive suppression), occur after an emotional response has been activated. This distinction is crucial, as each type of regulation carries different social, cognitive, and affective implications [7, 8].

Cognitive reappraisal, an antecedent-focused strategy, involves altering one’s perception of a potentially emotion-eliciting event to decrease its emotional impact. This strategy diminishes negative emotional experiences and physiological responses, and enhances the expression and experience of positive emotions, fostering closer relationships and greater personal well-being [7, 8]. In contrast, expressive suppression, a response-focused strategy, entails inhibiting the outward expression of emotions. While it does not alter the emotional experience itself, suppression increases physiological activation and can have detrimental effects on social interactions, personal relationships, and overall mental health, as it decreases the expression of both positive and negative emotions and is associated with greater depressive symptoms, lower self-esteem, and reduced optimism [7, 8].

In summary, the body of research led by Gross and his collaborators [6–8] highlights the differential effects of emotion regulation strategies. Cognitive reappraisal emerges as a more adaptive technique, given its positive outcomes on affect, relationships, and well-being, contrasting with the less advantageous impacts of expressive suppression.

### Neurobiological foundations of emotion regulation: bridging attachment styles and neural mechanisms

Understanding the neurobiological underpinnings of emotion regulation provides a crucial foundation for exploring how various attachment styles influence emotion regulation strategies. Functional magnetic resonance imaging (fMRI) studies have elucidated the roles of specific neuroanatomical structures in emotional processing, highlighting the ventral system (including the amygdala, insula, and anterior cingulate cortex) for emotion generation and detection [9–13], and the dorsal system (encompassing the dorsolateral prefrontal cortex, ventrolateral prefrontal cortex, and orbitofrontal cortex) for emotion regulation [14–23].

The interplay between these neural systems and attachment styles is pivotal in understanding emotion regulation. For instance, securely attached individuals, who typically exhibit more effective emotion regulation strategies, may have more robust neural communication between prefrontal regions and the amygdala, facilitating adaptive regulatory responses [19, 23, 24]. In contrast, individuals with insecure attachment styles (anxious, avoidant, and fearful) might display altered patterns of neural activation or connectivity that correlate with less effective emotion regulation strategies [13, 25, 26].

Recent studies have begun to map these differences in neural architecture and function to specific attachment styles. For example, Vrtička and colleagues [13, 26] demonstrated that secure attachment is associated with enhanced prefrontal cortex modulation of amygdala activity during emotion regulation tasks, facilitating cognitive reappraisal. Insecurely attached individuals, however, show distinct neural patterns that may undermine the effectiveness of such strategies, potentially due to less efficient prefrontal inhibition of the amygdala or less functional communication [25, 26]. These findings highlight the significance of neural mechanisms in the relationship between attachment styles and emotion regulation capabilities. This neural perspective enriches our understanding of why individuals with different attachment styles may exhibit varying efficacies in their use of emotion regulation strategies.

### Attachment theory

Attachment theory, proposed by Bowlby [27], explains the tendency of individuals to form intimate emotional bonds with specific persons as a fundamental aspect of human nature. This theory has gained strength over the years and applies to our entire lifespan, from birth to old age.

Attachment theory comprises several components, one of which is attachment behavior. Bowlby [28] refers to attachment behavior as any form of behavior that seeks closeness or the preservation of proximity with another individual perceived as more capable of coping with the world, providing a sense of security. This pursuit of proximity occurs primarily when a person is faced with emotionally stressful situations, danger, or threats, which are alleviated by accessing the care and comfort offered by the attachment figure we turn to, known as the attachment figure [28]. During infancy, parents or caregivers typically fulfill the role of the attachment figure, and in adolescence and adulthood, these bonds persist while also being complemented by new relationships [27, 29].

Early interactions influence the formation of what Bowlby [30] termed as Internal Working Models (IWMs). According to this model, through interaction with attachment figures, a mental representation of oneself and others is internalized [28]. This representation expresses expectations held about oneself and others and enables the anticipation, interpretation, and response to the behaviors of attachment figures [29]. In adulthood, attachment refers to the emotional bonds that individuals establish with others throughout their lives. These bonds have a significant impact on how individuals perceive, experience, and respond to interpersonal relationships [31].

Within the theory of adult attachment, a widely accepted notion is that adult attachment can be described along two dimensions or orientations: attachment anxiety and attachment avoidance, which are associated with the IWM of self and the IWM of others, respectively [32–34]. Attachment anxiety refers to the fear of abandonment in relationships and is rooted in a negative IWM of self. Individuals with high anxiety exhibit a strong need for approval and emotional dependency. They also tend to hyperactivate their attachment needs, which is reflected in the constant seeking of closeness and chronic frustration when perceiving a lack of response. On the other hand, attachment avoidance refers to discomfort with closeness, intimacy, and dependency, based on expectations of rejection (negative IWM of others). Individuals with high avoidance exhibit heightened self-sufficiency, reluctance to seek support, distrust in others, and a tendency to deactivate their own and others’ emotional needs [35, 36]. These orientations serve as filters through which relationships are interpreted and experienced.

Individuals who exhibit high levels of anxiety and avoidance in relationships are considered insecure in their attachment style [33], whereas those who experience low levels of anxiety and avoidance tend to be secure in their attachment. The combination of these dimensions allows for the identification of four attachment styles: secure (low anxiety and avoidance), anxious/preoccupied (high anxiety, low avoidance), dismissive-avoidant (low anxiety, high avoidance), and fearful-avoidant (high anxiety and avoidance) [33–35].

A robust body of research conducted in recent decades on adult attachment demonstrates that differences in attachment security/insecurity are consistently related to various indicators of individual and relational functioning [35]. Higher levels of attachment insecurity are associated with greater difficulties in emotional regulation [37–39] and a range of mental health problems [40–45].

In the measurement of adult attachment, the Experiences in Close Relationships (ECR) is the most widely used instrument for assessing adult attachment due to its robust psychometric properties tested in different contexts and cultures [35]. The Spanish version of the ECR has been validated in various Spanish-speaking populations [46, 47]. Recently, Guzmán-González et al. [48] proposed a brief version of the ECR consisting of 12 items (ECR-12), which was tested in diverse samples and demonstrated good psychometric properties. Reference values have also been provided [49].

While there is consensus that attachment security is more accurately described in dimensional terms rather than categorical terms [50], there are contexts where it can be useful to have preliminary information about the predominant attachment style within a group of individuals [49]. In this regard, the formation of groups of individuals categorized according to their attachment style within the context of studying emotional regulation could provide initial guidance for understanding how different attachment styles relate to the effective utilization of different emotional regulation strategies and identify patterns and potential difficulties for specific attachment styles.

Therefore, the decision to use a categorical rather than a dimensional approach in assessing attachment styles in this study was driven by the aim to provide clear, distinct profiles of emotional regulation strategies associated with each attachment style. While a dimensional approach offers a nuanced spectrum of attachment-related traits [50], a categorical model allows for the identification of patterns and potential difficulties unique to each attachment style. This method facilitates a clearer understanding of how different attachment styles might influence the effectiveness of specific emotion regulation strategies, thereby providing more targeted insights for both theoretical understanding and practical applications in clinical settings. This approach aligns with the concept of the malleability of attachment styles, considering that therapy could potentially modify maladaptive attachment patterns, further aiding in emotion regulation [51, 52].

### Emotional regulation and attachment styles

Several studies have established a relationship between an individual’s attachment style and the emotional regulation strategies they employ [13, 17, 35, 53, 54]. In a study conducted by Guzmán-González et al. [55], it was found that individuals with a secure attachment style experience fewer difficulties in emotional control, whereas those with high levels of anxiety experienced greater daily interference, lack of control, and emotional rejection. On the other hand, Henschel et al. [56] concluded that anxious attachment styles present difficulties in identifying and accepting emotions, impulsivity control, interference with goal-oriented behaviors, and difficulties in accessing emotional regulation strategies. According to Mikulincer and Shaver [31], individuals with an anxious attachment style tend to resort to hyperactivation strategies, which intensify negative social situations.

Regarding attachment avoidance, Guzmán-González et al. [55] found that individuals with high levels of avoidance experienced difficulties in attending to and recognizing their emotions. These findings align with the results obtained by Henschel et al. [56], who concluded that avoidant individuals exhibit greater difficulties in identifying emotions compared to those with a secure attachment style. Therefore, according to Mikulincer and Shaver [31], individuals with an avoidant attachment style tend to employ deactivation strategies, reducing the activation of the attachment system. This is in line with the findings of Vrticka et al. [26], who suggest that individuals with avoidant attachment predominantly utilize suppression as an emotional regulation strategy and generate less effective reappraisal strategies for regulating negative social emotions.

Moreover, individuals exhibiting a fearful attachment, characterized by high levels of both anxiety and avoidance, may encounter challenges in effectively regulating their emotions as a result of conflicting desires for closeness and fear of rejection or abandonment. Research on this particular attachment style is relatively limited due to its lower prevalence [13]. Collectively, these studies provide support for the existence of a relationship between attachment style characteristics and emotional regulation difficulties, emphasizing the distinctions between secure attachment, attachment avoidance, and attachment anxiety.

Gross’s process model of emotion regulation delineates the distinction between antecedent-focused and response-focused strategies [6]. Antecedent-focused strategies, such as situation selection or modification, are employed before emotional responses are fully generated, aiming to prevent the onset of negative emotions [17]. Insecure attachment styles influence the propensity to utilize these strategies differently [31]. For individuals exhibiting avoidant attachment, antecedent-focused strategies often involve preemptive measures to avoid emotional engagement or to minimize the impact of potential emotional stimuli. This includes strategies like situation selection, where individuals actively avoid circumstances that might evoke distress; situation modification, where they attempt to alter aspects of the environment to reduce potential stressors; and attentional deployment, such as distraction or concentration on non-emotional aspects of situations to avoid emotional engagement [31]. These strategies aim to preemptively manage emotional responses by controlling exposure to or the nature of emotional stimuli, thereby often circumventing the need for reappraisal or suppression. Similarly, individuals with anxious attachment may engage in a different set of antecedent-focused strategies that align with their heightened sensitivity to threat cues and their strong desire for closeness and reassurance. These strategies might include excessive reassurance-seeking, hyper-vigilance to signs of rejection or abandonment, or even preemptive expressions of distress to elicit support or attention from others [39]. While these strategies can exacerbate distress in the long term, they serve as initial attempts to regulate emotions by modifying the social environment or by seeking to influence the behavior of others.

However, when these primary, antecedent-focused strategies fail or are deemed insufficient, individuals may resort to secondary strategies, such as cognitive reappraisal and expressive suppression [17]. It is within this context that our study investigates these secondary strategies across different attachment styles. While cognitive reappraisal and suppression might be later options in the emotion regulation process, their effectiveness can provide valuable insights into the regulatory capabilities and limitations of individuals with insecure attachment, particularly in scenarios where primary antecedent-focused strategies are bypassed or ineffective [6].

Moreover, the precise relationship between the distinct emotional facets of non-interpersonal emotion regulation and the categorical model of adult attachment in Latin American samples remains poorly elucidated. Understanding how attachment styles relate to specific effectiveness in implementing emotion regulation strategies within this cultural context might provide valuable insights. Specifically, we aim to investigate the regulation of valence and arousal utilizing strategies such as expressive suppression and cognitive reappraisal. Here, effectiveness is defined as the ability to modulate emotional responses, specifically the reduction of unpleasant emotions, as evidenced by changes in self-reported valence and arousal levels.

In this study, we employed an emotion regulation task wherein participants were required to rate two dimensions of their emotional experience: valence, pertaining to the perceived pleasantness or unpleasantness of an event, and arousal, reflecting the perceived intensity of the emotion. Within our behavioral paradigm, all participants were instructed to actively engage in attending to, reappraising the emotional event (antecedent-focused regulation), or suppressing their emotional response (response-focused regulation) while being exposed to emotionally neutral or negative images. Subsequently, participants provided ratings of their emotional experience. Therefore, the effectiveness of implementing emotion regulation strategies was assessed by analyzing variations in valence and arousal scores associated with the experienced emotions.

In a previous study, we investigated the relationship between emotion regulation, attachment orientations from a dimensional perspective, and the effectiveness of different emotion regulation strategies [37]. The results revealed a significant association between attachment avoidance and arousal levels during the reappraisal condition. Specifically, individuals with higher levels of attachment avoidance experienced greater emotional intensity when implementing the cognitive reappraisal strategy, suggesting that individuals with higher attachment avoidance struggle to effectively downregulate intense emotions using cognitive reappraisal [31, 37, 57].

### The present study

The main objective of our research is to explore how different attachment styles, as defined in the categorical model of attachment, are associated with the effectiveness of specific emotion regulation strategies, namely cognitive reappraisal and expressive suppression. This objective involves assessing how individuals with different attachment styles (secure, anxious, avoidant, and fearful) differ in their ability to regulate emotions in terms of valence and arousal when employing these strategies.

We postulate that the four identified attachment styles exhibit distinct associations with performance in emotion regulation, encompassing both cognitive reappraisal and expressive suppression, within our emotion regulation task. Our objective is to characterize the effectiveness of the implementation of emotional regulation strategies according to the attachment style as per the categorical model of attachment proposed by Bartholomew and Horowitz [33]. To achieve this, we first evaluated the effectiveness of cognitive reappraisal and expressive suppression within each attachment style group. Subsequently, we compared these results across groups to understand the intergroup interactions.

The implications of our research are noteworthy in terms of advancing our comprehension of the interplay between attachment styles and emotional regulation. Furthermore, this knowledge can inform the development of interventions aimed at assisting individuals with insecure attachment in enhancing their emotional regulation skills. By shedding light on the specific challenges faced by individuals with diverse attachment styles, our research contributes valuable insights for the design of targeted therapeutic approaches tailored to their needs.

## Methods

### Participants

Ninety-Eight Chilean Latin-American adults (41 males, 53 females, 4 non-binary) aged between 18 and 58 years (mean age = 26.78; standard deviation (SD) = 8.86) were included in the data collection (Table 1). The sample size was calculated using G*Power 3.1.9.7 software (http://www.gpower.hhu.de/), considering the statistical one-way ANOVA test, an effect size of 0.5, an alpha value of 0.05, and a power of 0.95, obtaining an actual power of 0.95 [58]. All participants had normal or corrected-to-normal vision and reported no history of neurological or psychiatric disorders. The sample was recruited through a call for voluntary participation via the official digital platforms of the School of Psychology (Escuela de Psicología) and the Faculty of Humanities (Facultad de Humanidades) at the Universidad Católica del Norte, Antofagasta City, Chile. This was a non-probabilistic, purposive sample, targeting individuals over 18 years old who had been in a romantic relationship for at least six months prior to the study as inclusion/exclusion criteria. Three participants were excluded from the sample due to system crashes during their session.

**Table 1:** Descriptive statistics of the ECR-12 score

Participants	*n*	Age			Anxiety attachment ECR-12			Avoidance attachment ECR-12		
		M	SD	Mdn	M	SD	Mdn	M	SD	Mdn
Female	53	26.66	8.67	23.00	4.07	1.53	4.00	2.48	1.37	2.00
Male	41	27.46	9.44	23.00	3.28	1.33	3.17	2.38	0.95	2.14
Non-binary	4	21.25	1.50	22.00	3.83	1.74	3.58	4.29	0.84	4.14
Total	98	26.78	8.86	23.00	3.73	1.49	3.67	2.52	1.24	2.14

M = Mean; SD = Standard Deviation; Mdn = Median.

### Instruments

#### Experiences in close relationships questionnaire (ECR-12)

The ECR-12 questionnaire [34] was utilized in this study to evaluate adult attachment by assessing two dimensions: attachment anxiety and attachment avoidance. We employed the validated Chilean version [48], which consists of 12 items in Spanish. The questionnaire is composed of two subscales, each containing 6 items: attachment avoidance (e.g., “*I feel nervous when my partner becomes too emotionally close to me*”) and attachment anxiety (e.g., “*I constantly seek reassurance from my partner that they love me*”). Respondents rate their agreement with these statements on a 7-point Likert scale, ranging from (1) strongly disagree to (7) strongly agree. Higher scores on either subscale indicate greater levels of anxiety or avoidance attachment, respectively. The Chilean ECR-12 [48] has demonstrated excellent psychometric properties, maintaining the same reliability as the 36-item Chilean ECR version [47] and the original ECR [34]. The cut-off values for the Chilean population have been reported by Guzmán-González et al. [49].

#### Emotion regulation experimental paradigm

In order to evaluate the regulation of emotions, we employed an experimental task for emotional regulation, adapted from studies conducted by Oschner et al. [12], Vrticka et al. [26], Domic-Siede et al. [37] and Schlumpf et al. [59]. The task was programmed using Presentation Software® by Neurobehavioral Systems (Version 18.0, www.neurobs.com, Neurobehavioral Systems, Inc., Albany, CA). The stimuli used in the task were a collection of images sourced from the International Affective Picture System (IAPS) [60], which has demonstrated satisfactory psychometric properties in the Chilean context, specifically for sets 7 and 14 [61]. A total of 60 images were carefully selected, consisting of 45 emotionally negative images and 15 emotionally neutral images (Supplementary Table S1, Table S2). These images were presented to participants under three experimental conditions: ‘Natural’, ‘Reappraise’, and ‘Suppress’ [37].

All procedures were conducted in the *Laboratorio de Neurociencia Cognitiva* (Cognitive Neuroscience Laboratory) of the *Escuela de Psicología* (School of Psychology) at the Universidad Católica del Norte. In a single session, participants responded to the ECR-12 questionnaire, which was randomly completed either before or after the experimental task, lasting approximately 10 min. The experimental session itself took about 20 to 25 min.

Prior to the commencement of the experimental task, participants underwent a training session to familiarize themselves with the experimental setup and the objectives of each trial condition, which lasted about 7 to 10 min. The training session consisted of three blocks, with three trials in each condition. During the experiment, participants were instructed to assess various dimensions of their emotions while viewing emotional or neutral images. The experimenter provided visual aids and explained the task before its commencement, stating, “A picture image will be displayed on this monitor screen, which may evoke negative or neutral emotions. Before the image appears, you will be presented with one of three instructions: ‘Natural’, ‘Reappraise’, or ‘Suppress’.”

Under the ‘Natural’ instruction, participants were instructed to actively observe the picture and pay attention to their experienced emotions. They were encouraged to imagine themselves as part of the depicted situation in order to fully engage with the image. When the ‘Reappraise’ instruction appeared, participants were instructed to view the picture and attempt to diminish the emotional impact through cognitive reappraisal techniques. For example, they could imagine the scene as part of a movie with actors and makeup or envision a positive outcome for the scenario depicted in the image. This way, participants were trained in self-based or situation-based cognitive reappraisal strategies [12]. The self-based reappraisal strategies involved observing the images from a third-person perspective, without emotional involvement, or considering the images as fictional and not representative of real events. On the other hand, the situation-based reappraisal strategy involved envisioning an improvement in the observed situation. Finally, when the ‘Suppress’ instruction appeared, participants were instructed to view the image and regulate the evoked emotion by avoiding any outward expression of the emotion perceived.

Upon viewing each image in accordance with the assigned instruction (‘Natural’, ‘Reappraise’, or ‘Suppress’), participants provided ratings for the experienced emotion using a 1 to 7 Likert scale, adopting the Self-Assessment Manikin (SAM) [62] for valence and arousal dimensions. Valence ratings ranged from 1 (indicating unpleasant) to 7 (indicating highly pleasant), while arousal ratings ranged from 1 (indicating not arousing) to 7 (indicating highly arousing). Our SAM adaptation involved employing the picture-oriented instrument but modifying it to use seven specific manikins, accompanied by written instructions: “Indicate how much displeasure/pleasure the image you saw caused you” for valence rating, and “Indicate the intensity of your emotional response to the image you saw” for arousal rating. In order to respond, participants used a mouse restricted to horizontal movement (the mouse could only move along the x-axis) across the seven manikin figures representing valence. After selecting a manikin by clicking on it, the cursor appeared below in the arousal measure of SAM. Here again, the cursor could only move along the x-axis, and the display disappeared after a click within a manikin.

Following the completion of the task training, participants were asked to report on the strategies they employed to adhere to the given instructions. This question was done to ensure that participants clearly understood the task and the instructions provided.

**Fig. 1:**
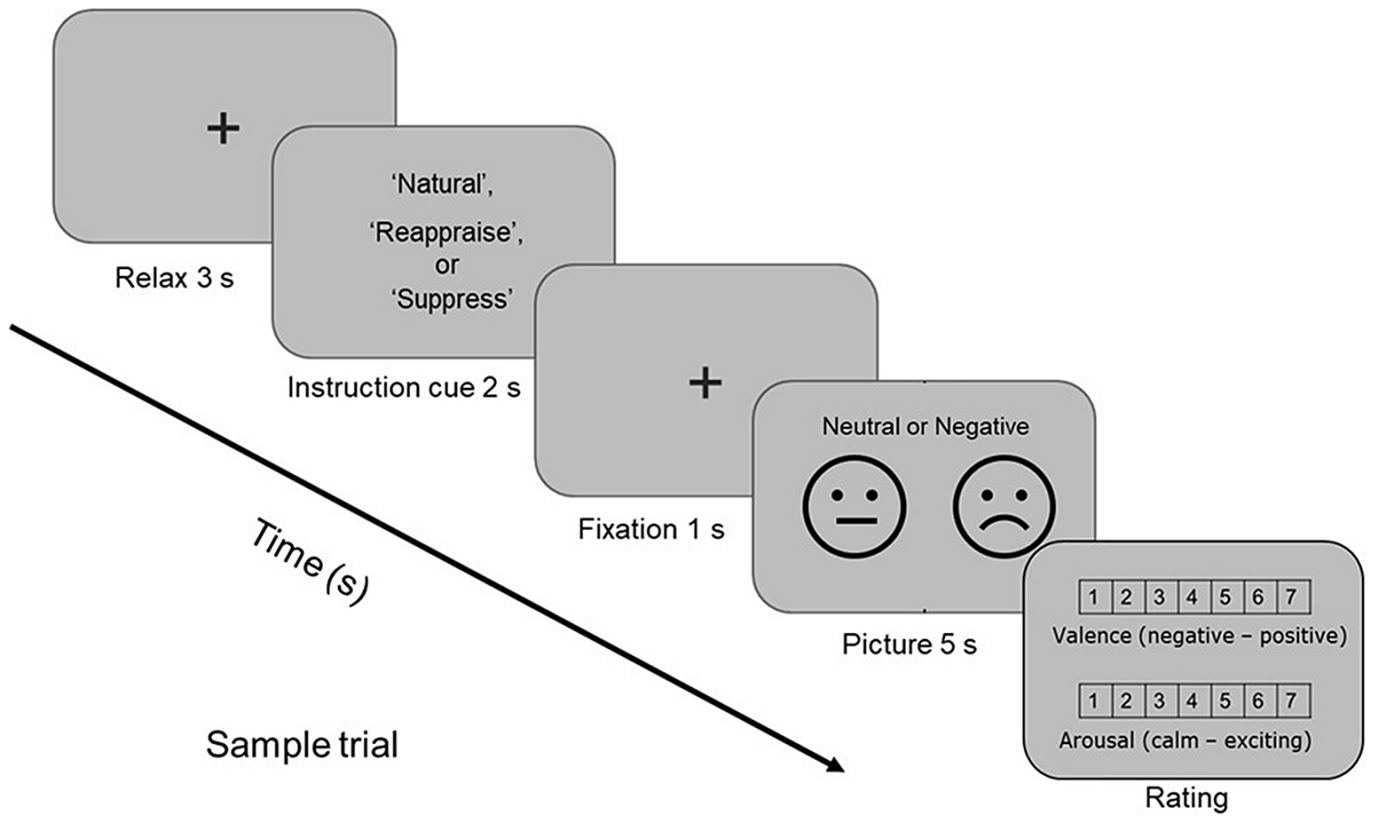
The emotion regulation task. Participants were instructed to actively observe either neutral or negative pictures in the ‘Natural’ condition. Alternatively, they were asked to regulate their anticipated emotional response to negative pictures using two distinct emotion regulation strategies: cognitive reappraisal or expressive suppression. Following each picture presentation, participants provided ratings for the subjective valence and arousal of the experienced emotion

Immediately after completing the training session, participants underwent the experimental task. The experimental session consisted of a randomized presentation of 12 blocks, each containing 5 pictures for the three conditions: ‘Natural’, ‘Reappraise’, and ‘Suppress’. Specifically, the ‘Natural’ condition included a total of 30 trials, with 15 negative and 15 neutral pictures. Subsequently, the ‘Natural’ condition was divided into two categories: ‘Natural-neu’ for natural neutral pictures and ‘Natural-neg’ for natural negative pictures. The ‘Reappraise’ and ‘Suppress’ conditions each comprised 15 trials with negative pictures only. It’s important to note that within this experimental framework, each block was uniformly dedicated to a single condition. This means that all 5 images within a given block corresponded to the same condition or instruction cue (e.g., all ‘Natural-neu’ or all ‘Suppress’), thereby ensuring the thematic consistency of the stimuli presented to participants during each segment of the task. Hence, in each block, the following sequence was followed, as depicted in Fig. 1: *i*) A gray background with a fixation cross “+” was displayed for 3 seconds, serving to orient the participant’s attention; *ii*) The task instruction indicating the condition block (‘Natural’, ‘Reappraise’, or ‘Suppress’) was presented for 2 seconds; *iii*) A cross fixation reappeared for 1 second at the center of the screen; *iv*) The picture (negative or neutral) was shown for 5 seconds; *v*) Finally, the Likert scales screen was presented, allowing participants to manually rate the valence and arousal of the experienced emotion using a computer mouse. After the last rating was marked, the trial sequence restarted, as stated in step *i*). This process was repeated until all blocks were completed. At the end of each block, a pause was displayed on the monitor before proceeding to the next block once the subject felt ready and pressed a button to continue.

It is noteworthy that, in our study, the assignment of images to the ‘Natural,’ ‘Reappraise,’ and ‘Suppress’ conditions were predetermined and consistent across all participants. Although the sequence of block presentation and the images within each block were randomized, the same specific images were always presented under their respective condition. This methodology ensured that each image was consistently associated with its respective experimental condition for all participants, thereby allowing for a standardized comparison of emotional responses across different conditions. The decision to structure the task into discrete blocks was predicated on the hypothesis that frequent transitions between distinct emotion regulation strategies might engender cognitive dissonance or confusion among participants, potentially compromising the integrity of the experimental outcomes.

In our experimental setup, the ‘Natural-neu’ and ‘Natural-neg” conditions were designed to assess arousal and valence of the emotions perceived in participants exposed to neutral and negative stimuli, respectively, without the use of specific emotion regulation strategies. The purpose of these conditions was to establish a baseline for the participants’ emotional responses to either neutral or negative stimuli. The ‘Natural-Neu’ condition functioned as a control for comparing responses to the emotionally charged negative images in the ‘Natural-neg’ condition. In turn, both these conditions provided a baseline against which the effectiveness of active emotion regulation strategies employed in other experimental conditions could be evaluated.

##### Calibration of IAPS picture sets

The International Affective Picture System (IAPS) was used to select images for the experimental conditions: ‘Natural’, ‘Reappraise’, and ‘Suppress’. The selection was based on previous research [37, 59, 63], and the valence and arousal ratings reported in the IAPS study [60]. The images chosen for the ‘Natural-neg’, ‘Suppress’, and ‘Reappraise’ conditions were equivalent in terms of valence and arousal, based on the findings reported in Domic-Siede et al. [37]. In contrast, the ‘Natural-neu’ condition showed significant differences. These selections were informed by the established suitability of these images for the emotional regulation paradigm, as outlined in [37].

### Data analyses

All data were analyzed using GraphPad Prism version 8 for Windows (GraphPad Software, La Jolla, California, USA, www.graphpad.com). Traditional descriptive statistics were calculated. The Shapiro-Wilk normality distribution test was conducted to determine whether parametric or non-parametric statistical hypothesis tests should be used [64]. This test was used on the data related to the emotion regulation task (valence and arousal for each condition), as well as on the anxiety and avoidance attachment scores (Supplementary Table S3 to S13). The levels of anxiety and avoidance attachment were compared using a Wilcoxon signed-rank test. Participants were classified into four attachment styles based on their scores on the ECR-12 questionnaire.

In alignment with the model proposed by Bartholomew and Horowitz [33], participants were classified into four attachment styles using scores from the ECR-12 questionnaire, employing the cut-off points reported in Guzmán-González et al. [49]. This study applied the z-score normalization method to establish reference values appropriate for the Chilean context based on a large sample of 6,779 adults. For individuals aged 29 or less, the cut-off points are as follows: for the anxiety dimension, a score equal to or greater than 4.4; for the avoidance dimension, a score equal to or greater than 2.5. For individuals aged 30 or older, the cut-off points are for the anxiety dimension, a score equal to or greater than 4.2, and for the avoidance dimension, a score equal to or greater than 2.9. This categorization into ‘secure,’ ‘anxious,’ ‘avoidant,’ and ‘fearful’ attachment styles (see Table 2) is based on these scores, allowing for a more nuanced understanding of attachment patterns, especially useful in clinical contexts. This classification facilitated the examination of performance on the emotion regulation task.

**Table 2:** Descriptive Statistics of the ECR-12 per Attachment Style Group

Attachment Styles		Age		Anxiety Attachment ECR-12		Avoidance Attachment ECR-12	
	*n*	M	SD	M	SD	M	SD
Secure	39	27.10	8.30	2.63	0.93	1.59	0.46
Anxious	16	26.13	9.48	5.35	0.83	1.76	0.43
Avoidant	24	26.29	8.23	3.12	0.68	3.71	1.05
Fearful	19	27.26	10.73	5.39	0.78	3.54	0.93
Total	98	26.78	8.86	3.73	1.49	2.52	1.24

M = Mean; SD = Standard Deviation

#### Within-group (attachment styles) analysis

Then, ANOVA or Friedman tests, as appropriate, were employed to analyze differences between emotion regulation effectiveness and control conditions for each attachment style group. The predictors in these models were the emotion regulation and control conditions (‘Natural’, ‘Reappraise’, ‘Suppress’), and the outcomes were the ratings of valence and arousal. Specifically, these tests assessed how participants with different attachment styles (secure, anxious, avoidant, fearful) responded in terms of valence and arousal across the different emotion regulation conditions to evaluate the effectiveness or performance of using emotion regulation strategies (cognitive reappraise and expressive suppression). The choice between ANOVA and the Friedman test depended on the data distribution. Finally, post-hoc tests with corrections for multiple comparisons were applied. Specifically, The Dunn and Tukey multiple comparison tests were used as follow-ups to the ANOVA or Friedman tests to conduct pairwise comparisons between the different conditions within each attachment style group. These tests helped to identify specifically which conditions differ from each other. The Dunn test is typically used as a follow-up to the Friedman test for non-parametric data, whereas the Tukey test is a common post-hoc test following ANOVA for parametric data. This approach allowed for a detailed exploration of the differences between the specific conditions (‘Natural,’ ‘Reappraise,’ ‘Suppress’) and provided insights into how these conditions impact emotional regulation effectiveness across different attachment styles.

#### Intergroup analysis

Following the within-group analysis, an intergroup analysis was conducted using the Kruskal-Wallis test. This analysis aimed to study differences in effectiveness in reducing displeasure or arousal when using either the ‘Reappraise’ or ‘Suppress’ strategies among the four attachment styles. The Kruskal-Wallis test was selected due to its suitability for comparing more than two groups in a non-parametric distribution. Additionally, a Dunn test was implemented for multiple comparisons following the Kruskal-Wallis test.

Furthermore, a separate Dunn test was conducted to compare the valence and arousal ratings of each attachment style group against a ‘Natural-negative’ condition. This condition was composed of all the values of the total sample under the ‘Natural’ condition with negative pictures. This comparison provided insight into how the emotion regulation strategies influenced emotional responses compared to a baseline natural response to negative stimuli.

Following the aforementioned analyses, we further implemented a two-way ANOVA. This analysis was specifically designed to study the interactions between the emotion regulation strategies (reappraise and suppression) and the attachment styles in terms of their effects on valence and arousal. Thus, two separate two-way ANOVAs were conducted: one for valence and the other for arousal. The two-way ANOVA approach allowed us to examine whether the impact of the reappraise and suppression strategies on emotional responses was consistent across the different attachment styles, or whether there were unique interactions between these factors. To further study the interactions and main effects revealed by the two-way ANOVAs, we employed the Sidak post-hoc test. This test was chosen for its ability to control the Type I error rate effectively when conducting multiple comparisons.

Effect sizes were calculated using rank-biserial correlation and Cohen’s d values to quantify the magnitude of the observed differences.

## Results

**Fig. 2:**
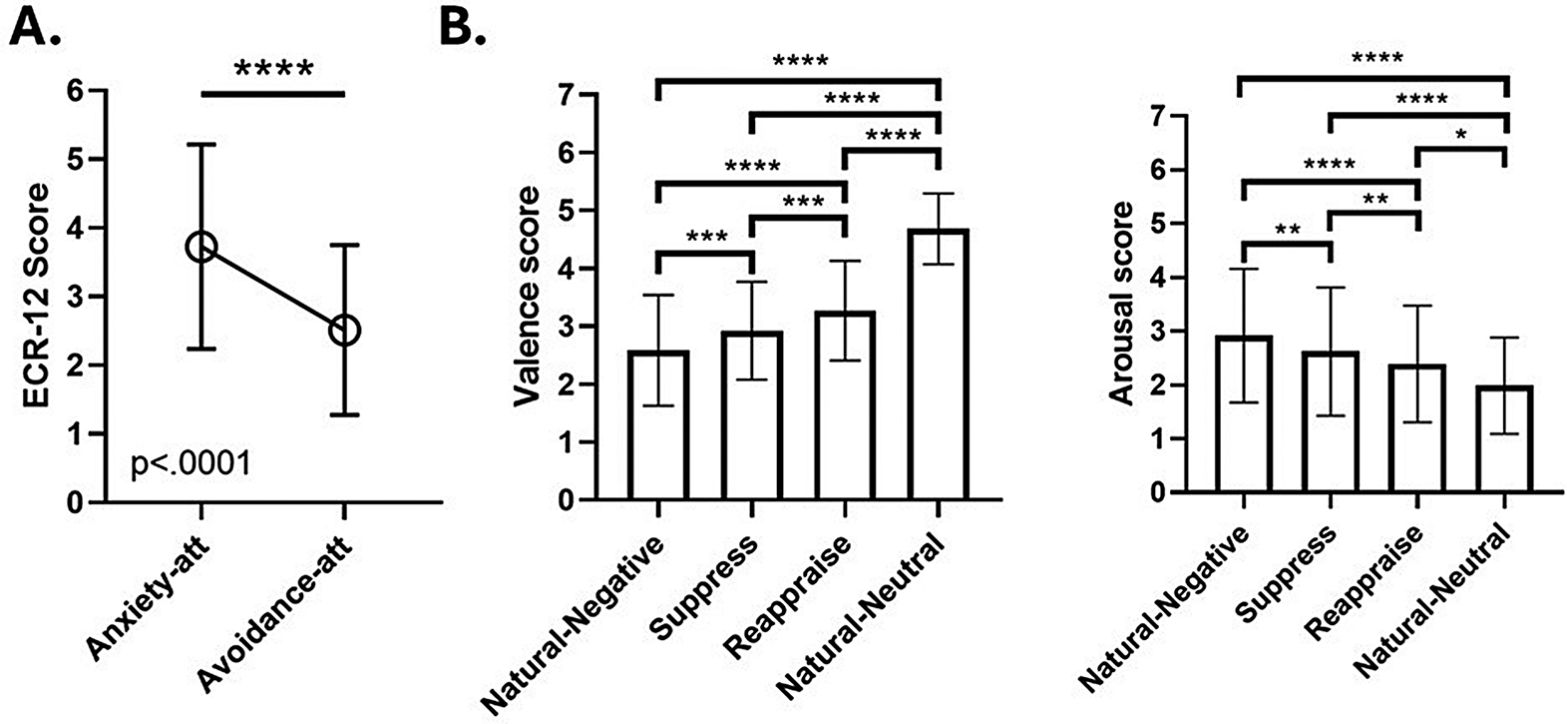
Attachment Dimensions and Emotion Regulation Performance. Total participants showed higher levels of anxiety attachment than avoidance attachment (**A**). The figure shows significant differences between the ratings in valence (left) and arousal (right) (**B**) when comparing ‘Natural-Neutral’ to the rest of the conditions for all participants. Significant differences were observed in the ratings of valence and arousal between the ‘Natural-Negative’ condition and both the ‘Reappraise’ and ‘Suppress’ conditions. Moreover, the ‘Reappraise’ condition (when participants use cognitive reappraisal strategy) was significantly more successful than the ‘Suppress’ condition (implementing expressive suppression) in reducing levels of displeasure and arousal, indicating that cognitive reappraisal was a more effective strategy than expressive suppression for regulating emotions. The numbers inside the bars are the mean scores. Anxiety-att = Anxiety Attachment; Avoidance-att = Avoidance Attachment. Error Bars indicate Standard Deviation

The analysis of ECR-12 of all participants revealed that anxiety attachment levels were higher than avoidance attachment levels, with a median difference of -1.155 (Wilcoxon signed-rank test: W = -3357, *p* < .0001, 96.66% CI [-1.83, -0.64], rank-biserial correlation = -0.692). The negative median difference indicates that anxiety attachment scores were generally higher than avoidance attachment scores among the participants. The strong effect size, suggested by the rank-biserial correlation, indicates that these differences were consistent and substantial across the sample (Fig. 2A). The purpose of comparing anxiety and avoidance scores was to evaluate the relative levels of these two dimensions of attachment within the sample. This within-person level comparison aimed to understand how these attachment dimensions manifested in the participant pool.

As expected, when analyzing the performance on the emotion regulation task of all participants, significant differences can be observed between conditions when rating valence (Q = 219.4; *p* < .0001) and arousal (Q = 110.9; *p* < .0001) using the Friedman test to assess variance (Fig. 2B), and then Dunn’s multiple comparison tests were performed to compare the rating values of valence and arousal.

For valence ratings, there are significant differences between the ‘Natural-Neutral’ condition and all other conditions: ‘Natural-Negative’ (*d* = -2.620; rank sum difference = -255.0; 95% CI [-3.001, -2.238]; p < .0001), ‘Suppress’ (*d* = -2.385; rank sum difference = -178.0; 95% CI [-2.751, -2.019]; p < .0001), and ‘Reappraise’ (*d* = -1.896; rank sum difference = -107.0; 95% CI [-2.233, -1.558]; p < .0001), with the ‘Natural-Neutral’ condition showing lower levels of displeasure, as expected. Among the other conditions, it is observed that in the ‘Reappraise’ condition, participants were able to significantly reduce their levels of displeasure compared to the reference condition, which is the ‘Natural-Negative’ condition (*d* = -1.896; rank sum difference = -148.0; 95% CI [-1.045, -0.465]; *p* < .0001). Although participants also managed to reduce their levels of displeasure in the ‘Suppress’ condition when compared to ‘Natural-Negative’ (*d* = -0.376; rank sum difference = -77.00; 95% CI [-0.658, -0.093]; p = .0001), the use of reappraisal was superior to the suppressive expression strategy (*d* = -0.407; rank sum difference = -71.00; 95% CI [-0.689, -0.124]; p < .0001).

In the analysis of arousal levels, significant differences were observed between the ‘Natural-Neutral’ condition and all other conditions. Specifically, comparisons revealed that the ‘Natural-Neutral’ condition had significantly lower arousal levels than the ‘Natural-Negative’ condition (*d* = 0.929; rank sum difference = 176.0; 95% CI [0.565, 1.151]; p < .0001), ‘Suppress’ condition (*d* = 0.601; rank sum difference = 118.0; 95% CI [0.314, 0.887]; p < .0001), and ‘Reappraise’ condition (*d* = 0.407; rank sum difference = 52.00; 95% CI [0.124, 0.690]; p = .0241). This indicates a consistently lower level of arousal in the ‘Natural-Neutral’ condition.

Further, when comparing the impact of cognitive strategies, it was found that the ‘Reappraise’ condition led to a more pronounced reduction in arousal levels compared to the ‘Natural-Negative’ condition (*d* = 0.449; rank sum difference = 124.0; 95% CI [0.165, 0.732]; p < .0001). Similarly, the ‘Suppress’ condition also demonstrated a significant reduction in arousal compared to the ‘Natural-Negative’ condition (*d* = 0.242; rank sum difference = 58.00; 95% CI [-0.039, 0.523]; *p* = .0080). Notably, the decrease in arousal was more substantial when employing the reappraisal strategy compared to expressive suppression (*d* = 0.201; rank sum difference = 66.00; 95% CI [-0.080, 0.481]; *p* = .0016) (Fig. 2B). These findings are consistent with existing literature and further affirm the task’s effectiveness for detailed investigation [12, 26, 37, 57].

Subsequently, we examined differences in performance on an emotional regulation task by classifying participants according to their attachment styles based on their scores on the ECR-12 questionnaire. For our analysis, we utilized the cutoff points for the anxiety and avoidance dimensions, differentiated by age groups, as per the findings from a sample of 6779 participants reported in Guzmán-González et al. [49]. For the group aged 29 years or less, the cutoff point for the anxiety dimension was a score equal to or greater than 4.4 points, and for avoidance, it was equal to or greater than 2.5 points. Conversely, for participants aged 30 or older, the cutoff point for the anxiety dimension was a score equal to or greater than 4.2 points, and for avoidance, it was equal to or greater than 2.9 points. Using these cutoff points, we classified our participants into four attachment styles according to the Bartholomew and Horowitz model [33]. As a result, we identified 35 subjects with secure attachment, 22 with anxious attachment, 22 with avoidant attachment, and 32 with fearful attachment (Table 2).

### Secure attachment style

The analysis of variance for valence (Q = 75.88; *p* < .0001) and arousal (Q = 36.86; *p* < .0001) ratings in the secure attachment style group revealed significant differences using the Friedman test. Subsequently, Dunn’s multiple comparison tests were performed to compare the rating values of valence and arousal.

As expected, passively viewing neutral images (‘Natural-Neutral’ condition) showed lower levels of displeasure compared to other conditions (‘Natural-Negative’ vs. ‘Natural-Neutral’; *d* = -2.033, rank sum difference = -94.00; 95% CI [-2.579, -1.486]; *p* < .0001; ‘Suppress’ vs. ‘Natural-Neutral’; *d* = -0.298; rank sum difference = -66.50; 95% CI [-0.744, 0.149]; p < .0001; ‘Reappraise vs. Natural-Neutral’; *d* = -1.535; rank sum difference = -39.50; 95% CI [-2.040, -1.030]; p = .0032).

**Fig. 3:**
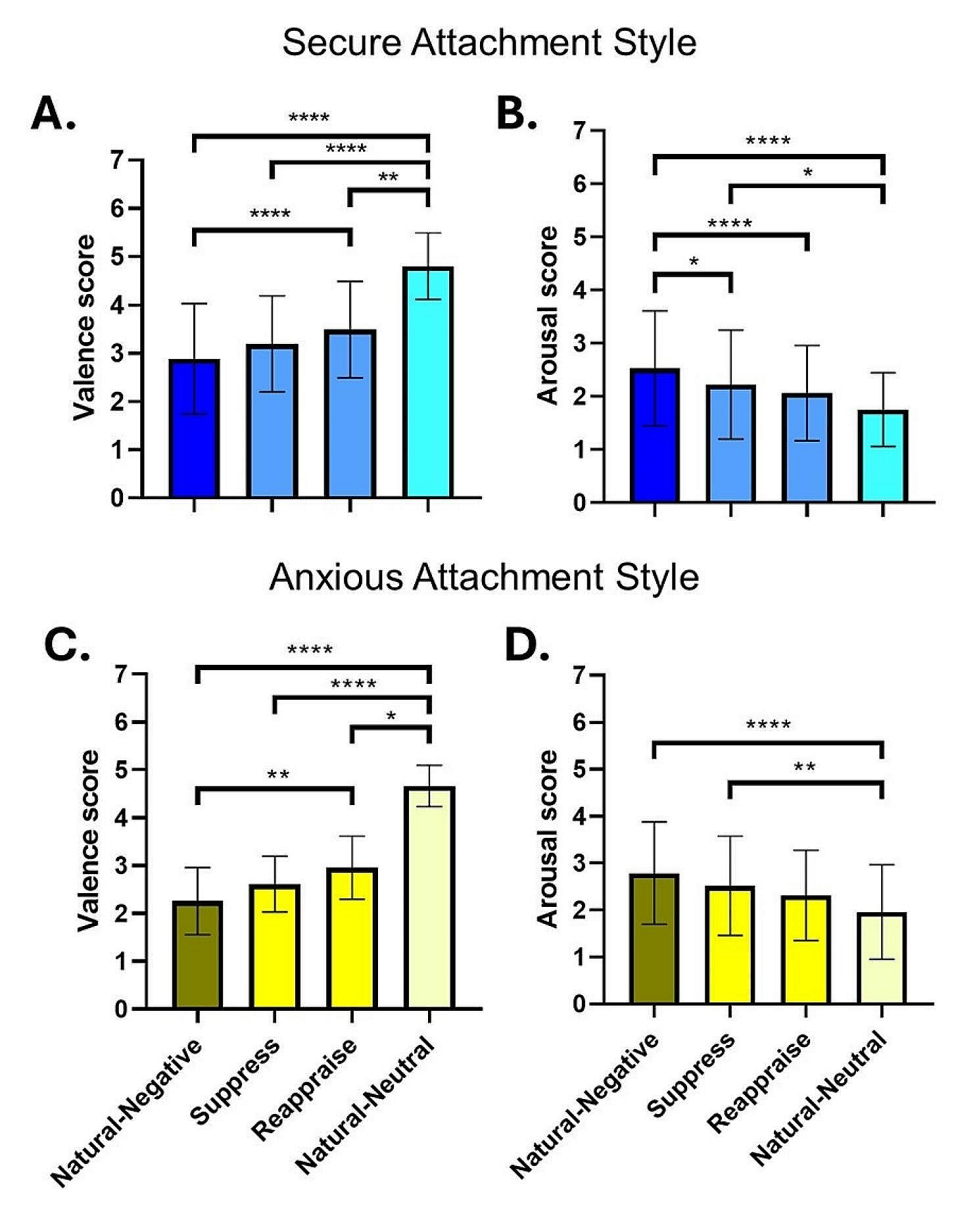
Emotion Regulation Performance of Secure and Anxious Attachment Styles Groups. **A**) Individuals with secure attachment styles effectively reduced their levels of displeasure by using the cognitive reappraisal strategy (‘Reappraise’ vs ‘Natural-Negative’). However, when employing the expressive suppression strategy, they experienced similar levels of displeasure as when simply viewing negative images (‘Suppress’ vs ‘Natural-Negative’), and these levels were significantly higher compared to viewing neutral images (‘Natural-Negative’ vs ‘Natural-Neutral’). **B**) In terms of arousal, there were no significant differences between viewing neutral images and using the cognitive reappraisal strategy. However, attempts to suppress emotions resulted in higher arousal levels, indicating ineffective emotion regulation through suppression. **C**) Participants with anxious attachment styles demonstrated the ability to effectively reduce their levels of displeasure by using the cognitive reappraisal strategy (Reappraise vs Natural-Negative). However, they were unable to decrease their displeasure when employing the expressive suppression strategy (‘Suppress’ vs ‘Natural-Negative’). **D**) Anxious attachment participants were unable to reduce their levels of arousal using either the cognitive reappraisal strategy (Reappraise vs Natural-Negative) or the expressive suppression strategy (‘Suppress’ vs ‘Natural-Negative’). However, while the ‘Suppress’ condition significantly differed from the ‘Natural-neutral’ condition, the ‘Reappraise’ condition did not. Error bars represent standard deviation. Asterisks denote levels of statistical significance, with *p < .05, ***p < .001, and ***p < .0001

Although there were no significant differences between the ‘Suppress’ and ‘Reappraise’ conditions (*d* = -0.298; rank sum difference = -27.00; 95% CI [-0.744, 0.149]; p = .1073), individuals with secure attachment styles demonstrated a notable ability to effectively decrease their levels of displeasure by employing the cognitive reappraisal strategy (‘Natural-Negative’ vs ‘Reappraise’; *d* = -0.288; rank sum difference = -54.50; 95% CI [-1.017, -0.112]; p < .0001). However, when employing the expressive suppression strategy, they exhibited similar levels of displeasure as when passively viewing negative images (‘Natural-Negative’ vs ‘Suppress’; *d* = -0.288; rank sum difference = -27.50; 95% CI [-0.735, 0.158]; p = .0952) (Fig. 3A).

Regarding arousal, the ‘Natural-Negative’ and ‘Natural-Neutral’ conditions exhibited significant differences (*d* = 0.856; rank sum difference = 64.00; 95% CI [0.392, 1.320]; *p* < .0001), as anticipated. Both suppressing expressions of emotions and reappraising emotional stimuli led to lower levels of arousal compared to passively viewing negative images (‘Natural-Negative vs. Suppress’; *d* = 0.290; rank sum difference = 33.00; 95% CI [-0.156, 0.736]; *p* = .0228; ‘Natural-Negative vs. Reappraise’; *d* = 0.470; rank sum difference = 51.00; 95% CI [0.020, 0.920]; *p* < .0001). However, there were no significant differences in arousal levels between passively viewing neutral images and employing the cognitive reappraisal strategy (‘Reappraise vs. Natural-Neutral’; *d* = 0.388; rank sum difference = 13.00; 95% CI [-0.060, 0.836]; *p* > .9999), while suppressing the expression of emotions showed higher levels emotional intensity (‘Suppress vs. Natural-Neutral’; *d* = 0.167; rank sum difference = 31.00; 95% CI [-0.278, 0.612]; *p* = .0393). There were no differences between ‘Suppress’ and ‘Reappraise’ (*d* = 0.167; rank sum difference = 18.00; 95% CI [-0.278, 0.612]; p = .6864) (Fig. 3B).

### Anxious attachment style

In the case of individuals categorized with an anxious attachment style, significant differences were observed in their responses to the emotion regulation task, as indicated by the analysis of variance for valence (Q = 41.26; *p* < .0001) and arousal (Q = 23.48; *p* < .0001) using the Friedman test. Subsequently, Dunn’s multiple comparison tests assessed the differences between conditions for both valence and arousal ratings.

For valence, those with an anxious attachment style demonstrated a notable reduction in displeasure when engaging in cognitive reappraisal, as shown by the comparison between the ‘Reappraise’ and ‘Natural-Negative’ conditions (‘Natural-Negative vs. Reappraise’; *d* = -1.019; rank sum difference = -25.50; 95% CI [-1.756, -0.282]; p = .0029), which is visually represented in Fig. 3C. In contrast, the attempt to decrease displeasure through expressive suppression did not yield a significant change, as the ‘Suppress’ condition did not differ significantly from the ‘Natural-Negative’ condition (‘Natural-Negative vs. Suppress’; *d* = -0.549; rank sum difference = -13.50; 95% CI [-1.255, 0.157]; *p* = .3871). As expected, the ‘Natural-Neutral’ condition was significantly different compared to the ‘Reappraise’ condition (‘Reappraise vs. Natural-Neutral’; *d* = -3.056; rank sum difference = -19.50; 95% CI [-4.077, -2.036]; *p* = .0455), the ‘Suppress’ condition (‘Suppress vs. Natural-Neutral’; *d* = -4.006; rank sum difference = -31.50; 95% CI [-5.207, -2.804]; *p* < .0001), and the ‘Natural-Negative’ condition (‘Natural-Negative vs. Natural-Neutral’; *d* = -4.116; rank sum difference = -45.00; 95% CI [-5.339, -2.892]; *p* < .0001). However, there were no significant differences between ‘Suppress’ and ‘Reappraise’ (‘Suppress vs. Reappraise’; *d* = -0.550; rank sum difference = -12.00; 95% CI [-1.256, 0.156]; p = .6021).

With respect to arousal, individuals with anxious attachment did not exhibit significant reductions when applying the instructed strategies of cognitive reappraisal or expressive suppression. The ‘Reappraise’ condition did not significantly differ from the ‘Natural-Negative’ condition in terms of arousal (‘Natural-Negative vs. Reappraise’; *d* = 0.464; rank sum difference = 19.00; 95% CI [-0.238, 1.166]; *p* = .0557), nor did the ‘Suppress’ condition (‘Natural-Negative vs. Suppress’; *d* = 0.253; rank sum difference = 5.00; 95% CI [-0.443, 0.949]; *p* > .9999), as depicted in Fig. 3D. However, only the strategy of expressive suppression differed significantly from the ‘Natural-Neutral’ condition, showing higher levels of emotional intensity (‘Suppress vs. Natural-Neutral’; *d* = 0.543; rank sum difference = 27.00; 95% CI [-0.163, 1.248]; *p* = .0013; ‘Reappraise vs. Natural-Neutral’; *d* = 0.360; 95% CI [-0.339, 1.058]; *p* = .4504). However, the ‘Suppress’ condition was not different from the ‘Reappraise’ condition (‘Suppress vs. Reappraise’; *d* = 0.203; rank sum difference = 14.00; 95% CI [-0.492, 0.898]; p = .3314). As expected, the ‘Natural-Negative’ condition showed higher levels of arousal compared to the ‘Neutral condition’ (‘Natural-Negative vs. Natural-Neutral’; *d* = 0.793; rank sum difference = 32.00; 95% CI [0.073, 1.513]; p < .0001).

### Avoidant attachment style

The group with avoidant attachment style showed significant differences in the analysis of variance for valence (F = 54.45; *p* < .0001) and arousal (Q = 27.63; *p* < .0001) ratings, using a One-way ANOVA and the Friedman test, respectively. Subsequently, Tukey’s multiple comparison tests were conducted to compare the rating values of valence between conditions, and Dunn’s multiple comparison tests were used for arousal.

**Fig. 4:**
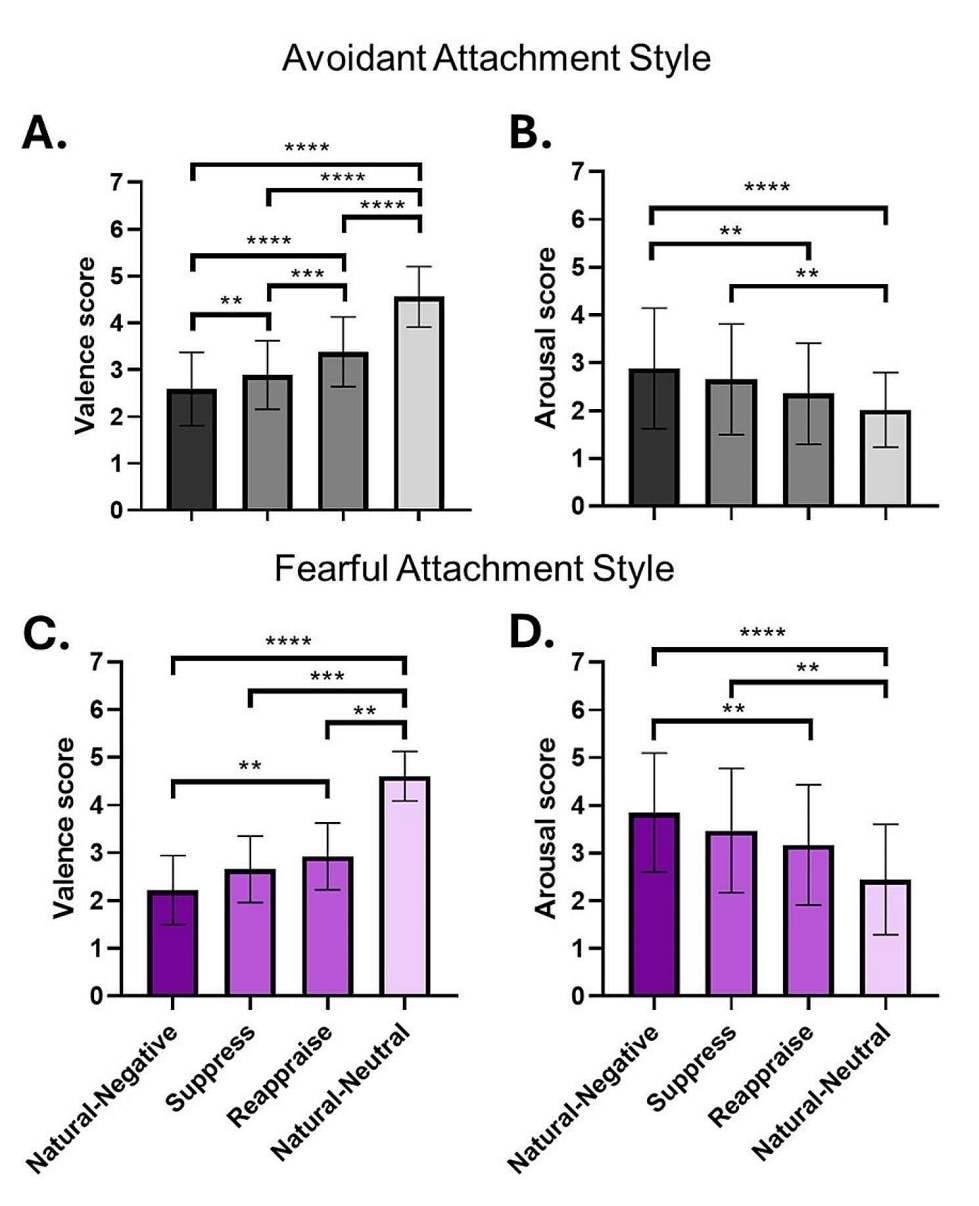
Emotion Regulation Performance of Avoidant and Fearful Attachment Styles Groups. **A**) Individuals with an avoidant attachment style were able to decrease their displeasure by using cognitive reappraisal (‘Reappraise’ vs. ‘Natural-Negative’) and expressive suppression (‘Suppress’ vs. ‘Natural-Negative’) as emotion regulation strategies. However, when attempting to suppress their emotional expression, they experienced higher levels of displeasure compared to the ‘Reappraise’ condition. **B**) In contrast, these individuals experienced difficulties in reducing arousal using expressive suppression, but their levels of arousal significantly were reduced under the ‘Reappraise’ condition. **C**) Individuals with a fearful attachment style successfully reduced both displeasure and **D**) arousal by employing cognitive reappraisal. Significance levels are denoted as follows: **p < 0.01, ***p < 0.001, ****p < 0.0001. The error bars represent standard deviations

Individuals in this group were able to reduce their levels of displeasure not only by using cognitive reappraisal (‘Natural-Negative vs. Reappraise’; *d* = -1.040; rank sum difference = -0.7943; 95% CI [-1.178, -0.4105]; *p* < .0001) but also by employing expressive suppression as an emotional regulation strategy (‘Natural-Negative vs. Suppress’; *d* = -0.399; rank sum difference = -0.3026; 95% CI [-0.4999, -0.1054]; *p* = .0016) (Fig. 4A). However, reappraisal was more effective than suppression in reducing levels of displeasure (‘Suppress vs. Reappraise’; *d* = -0.666; rank sum difference = -0.4917; 95% CI [-0.7639, -0.2195]; *p* = .0003). As expected, the ‘Natural-Neutral’ condition showed significantly lower levels of displeasure compared to the rest of the conditions (‘Natural-Negative vs. Natural-Neutral’; *d* = -2.750; rank sum difference = -1.972; 95% CI [-2.583, -1.361]; *p* < .0001; Reappraise vs. Natural-Neutral; *d* = -1.693; rank sum difference = -1.178; 95% CI [-1.719, -0.6364]; *p* < .0001; ‘Suppress vs. Natural-Neutral’; *d* = -2.420; rank sum difference = -1.669; 95% CI [-2.255, -1.084]; *p* < .0001).

However, concerning arousal, these individuals experienced high levels of arousal when attempting to suppress their emotions since there was no difference between suppression and the ‘Natural-Negative’ condition (‘Natural-Negative vs. Suppress’; d = -0.250; rank sum difference = 8.000; 95% CI [-0.816, 0.316]; *p* > .9999) (Fig. 4B). Consistently, there was a significant difference in arousal levels when comparing suppression to the natural neutral condition (‘Suppress vs. Natural-Neutral’; d = -1.690; rank sum difference = 33.50; 95% CI [-2.256, -1.124]; *p* = .0011). In contrast, when comparing suppression to reappraisal, the difference in arousal was less pronounced and not statistically significant (‘Suppress vs. Reappraise’; *d* = -0.444; rank sum difference = 20.50; 95% CI [-1.009, 0.122]; *p* = .1314). However, when reappraisal was compared to the natural neutral condition, the difference in arousal was not significant (‘Reappraise vs. Natural-Neutral’; *d* = -1.266; rank sum difference = 13.00; 95% CI [-1.832, -0.700]; *p* = .8766). Moreover, when reappraising, individuals showed a decrease in arousal levels compared to the ‘Natural-Negative’ condition (‘Natural-Negative vs. Reappraise’; d = -0.682; rank sum difference = 28.50; 95% CI [-1.248, -0.116]; *p* = .0086). As expected, the ‘Natural-Neutral’ condition exhibited lower levels of arousal compared to the ‘Natural-Negative’ condition (‘Natural-Negative vs. Natural-Neutral’; *d* = -1.877; rank sum difference = 41.50; 95% CI [-2.443, -1.312]; *p* < .0001).

### Fearful attachment style

Lastly, individuals with a fearful attachment style exhibited significant differences in the analysis of variance for valence (Q = 46.55; *p* < .0001) and arousal (Q = 26.85; *p* < .0001) ratings using The Friedman test. Subsequently, Dunn’s multiple comparison tests were conducted to compare the rating values of valence and arousal between conditions.

This group successfully reduced their levels of displeasure and arousal using the instructed strategy cognitive reappraisal when compared to the ‘Natural-Negative’ condition (‘Natural-Negative vs. Reappraise’; Valence: *d* = -0.991; rank sum difference = -26.00; 95% CI [-1.626, -0.355]; *p* = .0065; Arousal: *d* = 0.540; rank sum difference = 25.50; 95% CI: [-0.096, 1.176]; *p* = .0081). While expressive suppression did not effectively reduce their levels of displeasure (‘Natural-Negative vs. Suppress’; *d* = -0.612; rank sum difference = -20.50; 95% CI [-1.248, 0.024]; *p* = .0600) nor arousal (‘Natural-Negative vs. Suppress’; *d* = 0.297; rank sum difference = 12.00; 95% CI [-0.339, 0.933]; *p* = .7895). Interestingly, reappraise did not show differences only between arousal levels compared to the ‘Natural-Neutral’ condition (‘Reappraise vs. Natural-Neutral’; *d* = 0.600; rank sum difference = 13.00; 95% CI [-0.035, 1.236]; *p* = .6141), whereas valence showed significant differences when compared between these two conditions (‘Reappraise vs. Natural-Neutral’; *d* = -2.727; rank sum difference = -27.50; 95% CI [-3.363, -2.091]; *p* = .0033). On the other hand, expressive suppression showed significant differences in valence (‘Suppress vs. Natural-Neutral’; *d* = -3.169; rank sum difference = -33.00; 95% CI [-3.805, -2.533]; *p* = .0002) and arousal (‘Suppress vs. Natural-Neutral’; *d* = 0.831; rank sum difference = 13.00; 95% CI [0.195, 1.467]; *p* = .6141) when compared to the ‘Natural-Neutral’ conditions.

The use of neither of the two strategies showed differences when we compared them in terms of valence (‘Suppress vs. Reappraise’; *d* = -0.387; rank sum difference = -5.50; 95% CI [-1.023, 0.249]; *p* > .9999) and arousal (‘Suppress vs. Reappraise’; d = 0.233; rank sum difference = 13.50; 95% CI [-0.403, 0.868]; *p* = .5389). As expected, the ‘Natural-Neutral’ condition and ‘Natural-Negative’ conditions showed values of valence (‘Natural-Negative vs. Natural-Neutral’; *d* = -3.781; rank sum difference = -53.50; 95% CI [-4.417, -3.146]; *p* < .0001) and arousal (‘Natural-Negative vs. Natural-Neutral’; d = 1.165; rank sum difference = 38.50; 95% CI [0.530, 1.801]; *p* < .0001) significantly different (Fig. 4C and D).

### Intergroup results

The intergroup analysis investigated the effectiveness of emotion regulation strategies—‘Reappraise’ and ‘Suppress’—across different attachment styles for modifying emotional valence and arousal. The Kruskal-Wallis test revealed significant differences among the attachment style groups in their response to these strategies. Post-hoc Dunn tests were then conducted to delineate the specific group differences.

**Fig. 5:**
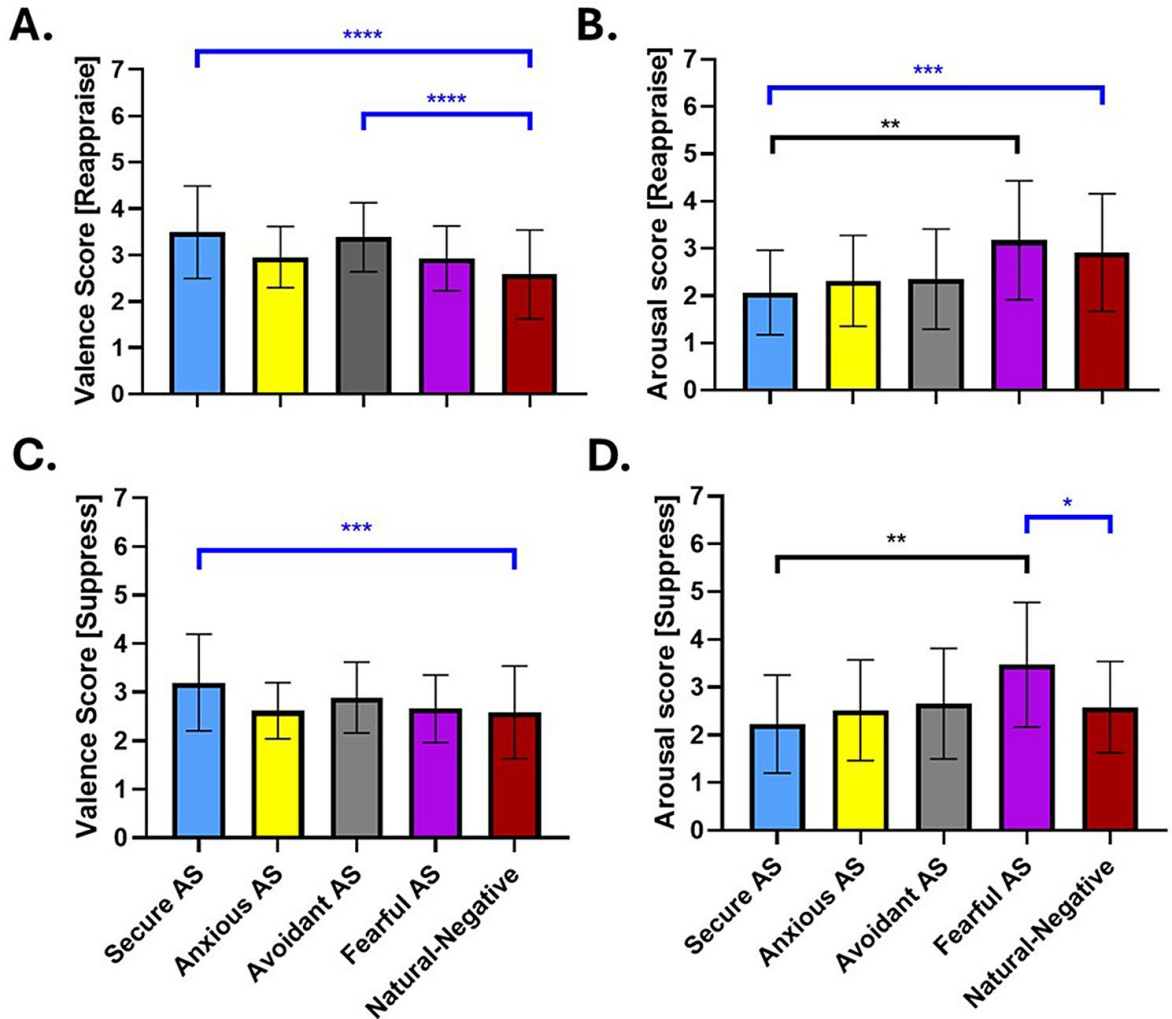
Differential Impact of Emotion Regulation Strategies Across Attachment Styles. This figure illustrates the differential effects of emotion regulation strategies, ‘Reappraise’ and ‘Suppress,’ on emotional valence and arousal across various attachment styles, as well as in comparison to a natural negative emotional context. Panels **A** and **B** represent the impact of the ‘Reappraise’ strategy on valence and arousal scores, respectively, while panels **C** and **D** depict the same for the ‘Suppress’ strategy. The Kruskal-Wallis test was employed to assess the intergroup effectiveness of the ‘Reappraise’ and ‘Suppress’ strategies in modulating displeasure (valence) and physiological arousal across the four attachment styles: Secure Attachment Style (Secure AS), Anxious Attachment Style (Anxious AS), Avoidant Attachment Style (Avoidant AS), and Fearful Attachment Style (Fearful AS). Statistical significance is indicated by black asterisks, which denote where the Dunn post-hoc test revealed significant differences between the attachment style groups in response to the regulation strategies. Blue asterisks indicate the results of a separate Dunn test comparing the emotional responses of each attachment style group to the ‘Natural-negative’ condition, which served as a baseline. This condition aggregates the responses of the entire sample to naturally experienced negative emotions without any regulation strategy applied. The aim was to contextualize the effects of ‘Reappraise’ and ‘Suppress’ strategies against the natural emotional response to negative stimuli. Significance levels are denoted as follows: *p < .05, **p < .01, ***p < .001, ****p < .0001. The error bars represent standard deviations

For the ‘Reappraise’ strategy, as depicted in Fig. 5A (H = 7.317; *p* = .0625), there was no differences in valence scores when comparing between AS groups (Supplementary Table S14). In contrast, we found differences across AS for arousal during ‘Reappraise’ (H = 12.76; *p* = .0052). The differences were found between Secure AS and Fearful AS where Secure AS were more effective in reducing arousal levels compared to Fearful AS group (Secure AS vs. Fearful AS; *d* = -1.080; mean rank difference = -28.35; 95% CI [-1.696, -0.4640]; *p* = .0022). There were no differences in arousal scores in other groups comparison (Fig. 5B; Supplementary Table S15).

Furthermore, we implemented the Dunn test to compare each AS group against the aggregate ‘Natural-Negative’ condition, which served as a baseline. This baseline condition encompassed responses from the entire sample experiencing negative emotions naturally, without the application of any regulation strategy. The purpose of this comparison was to evaluate the efficacy of the ‘Reappraise’ and ‘Suppress’ strategies in mitigating valence displeasure and arousal. Our findings indicate that the valence scores during ‘Reappraise’ for both the Secure AS (Natural-Negative vs. Secure AS; *d* = 0.942; mean rank difference = -54.78; 95% CI [0.564, 1.320]; p < .0001) and Avoidant AS (Natural-Negative vs. Avoidant AS; *d* = 0.871; mean rank difference = -59.82; 95% CI [0.487, 1.255]; p < .0001) were significantly higher in relation to the ‘Natural-Negative’ condition, suggesting a significant decrease in displeasure (Fig. 5A, marked with blue asterisks). Notably, the Secure AS group was distinguished as the sole group exhibiting a substantial reduction in arousal levels when compared to the Natural-Negative condition (‘Natural-Negative’ vs. Secure AS; *d* = -0.737; mean rank difference = 45.53; 95% CI [-1.060, -0.413]; *p* = .0003), as depicted in Fig. 5B (highlighted with blue asterisks). Other AS groups did not show differences in valence or arousal when compared to the ‘Natural-Negative’ condition (Supplementary Table S16, S17).

In contrast, the ‘Suppress’ strategy showed a different pattern of effectiveness. Valence scores (H = 6.832; *p* = .0774; Fig. 5C) did not vary as significantly among the groups (Supplementary Table S18). Arousal scores (H = 13.17; *p* = .0043; Fig. 5D) revealed that the Suppress strategy led to a significant reduction in arousal for the Secure AS group compared to the Fearful AS group (Secure AS vs. Fearful AS; *d* = -1.109; mean rank difference = -28.74; 95% CI [-1.705, -0.514]; *p* = .0018). There were no significant differences in arousal among other AS groups (Supplementary Table S19).

When comparing each AS group to the ‘Natural-Negative’ baseline condition, we found that Secure AS showed higher levels of valence, meaning a significant reduction in displeasure when suppressing (Fig. 5C; Supplementary Table S20; ‘Natural-Negative’ vs. Secure AS; *d* = 0.636; mean rank difference = -39.75; 95% CI [0.258, 1.013]; *p* = .0009). Regarding arousal, interestingly, Fearful AS exhibited higher levels of arousal compared to the ‘Natural-Negative’ condition (Fig. 5D; Supplementary Table S21; ‘Natural-Negative’ vs. Fearful AS; *d* = 0.874; mean rank difference = -38.44; 95% CI [0.269, 1.479]; *p* = .0273).

In the subsequent phase of our intergroup analysis, we applied two separated two-way ANOVA analysis, each dedicated respectively to valence and arousal. This analytical framework was designed to elucidate the interplay between emotion regulation strategies—namely, reappraisal and suppression—and the four AS groups.

In our analysis focusing on valence, the two-way ANOVA revealed significant main effects for both the AS factor (F(3, 188) = 5.567, *p* = .0011), and the emotion regulation strategies factor (F(1, 188) = 7.801, *p* = .0058). However, the interaction between these factors was not significant (F(3, 188) = 0.1740, *p* = .9139). Specifically, the least squares (LS) mean for valence was higher for reappraisal (3.188) compared to suppression (2.838), with a mean difference of 0.3500 (95% CI [0.1028, 0.5972]).

In the corresponding analysis of arousal, the two-way ANOVA indicated a significant main effect for the AS factor (F(3, 188) = 10.34, *p* < .0001), while the emotion regulation strategies factor and the interaction between the two factors were not significant (Emotion Regulation Strategies: F(1, 188) = 2.227, *p* = .1373; interaction: F(3, 188) = 0.05998, *p* = .9807). The LS mean for arousal under reappraisal (2.475) was lower compared to suppression (2.717), with a mean difference of -0.2420 (95% CI [-0.5619, 0.07789]).

These results suggest that while emotion regulation strategies and attachment styles independently influence valence and arousal, their interaction does not significantly impact these emotional responses in our sample.

**Fig. 6:**
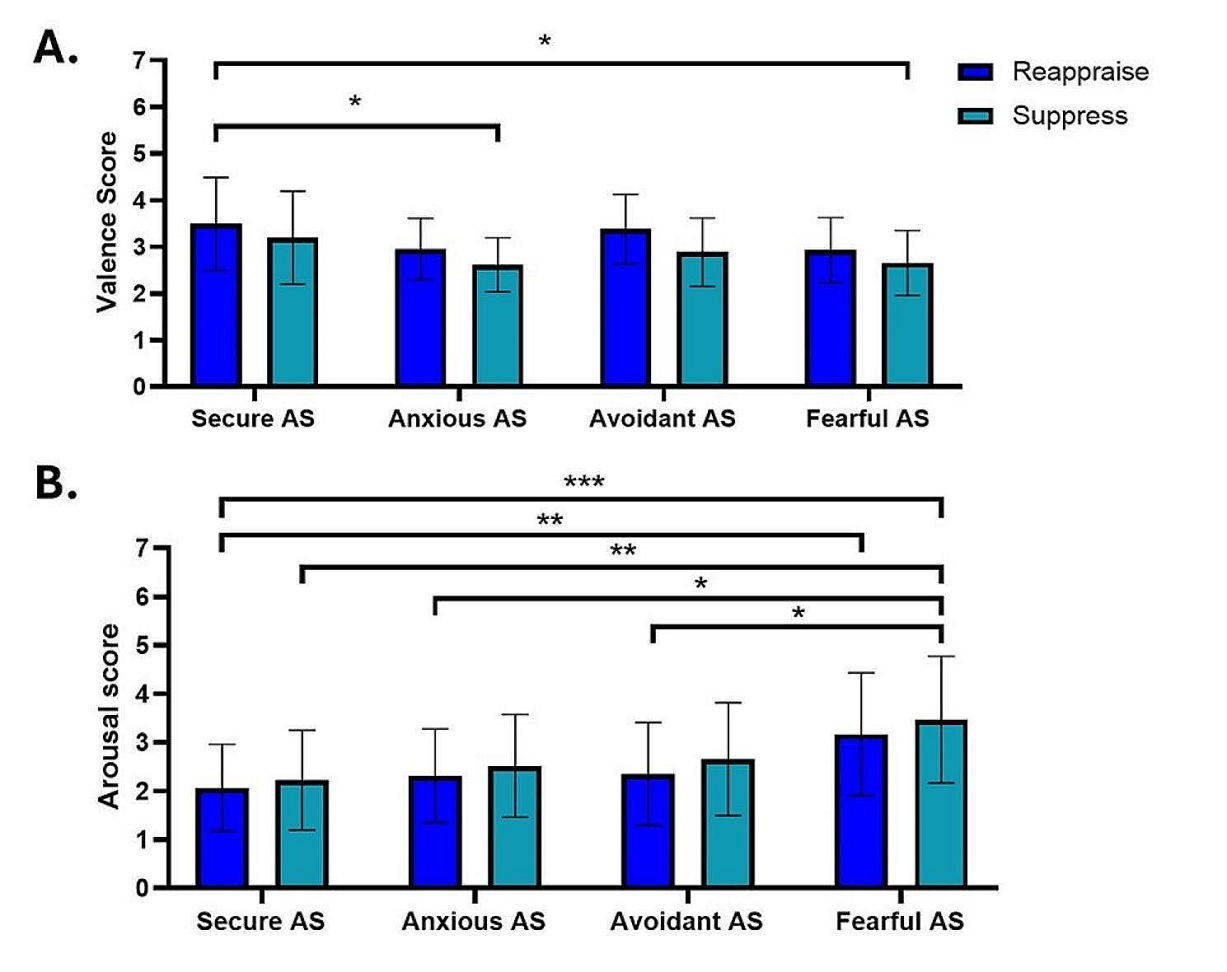
Differential Impact of Emotion Regulation Strategies Across Attachment Styles. **A**) The bar graph illustrates the valence scores for Secure, Anxious, Avoidant, and Fearful Attachment Styles (AS) under conditions of Reappraisal (blue) and Suppression (light blue). The Secure AS group showed significantly higher valence during Reappraisal compared to the Anxious AS group during Suppression and compared to the Fearful AS group during Suppression. **B**) The bar graph represents the arousal scores for the same attachment styles under the two emotion regulation strategies. Notably, the Secure AS group exhibited significantly lower arousal levels during Reappraisal compared to Fearful AS during both Reappraisal and Suppression. A significant reduction in arousal was also observed for the Avoidant AS group during Reappraisal when compared to the Fearful AS group during Suppression, as well as a marginal difference in arousal between the Anxious AS during Reappraisal and the Fearful AS during Suppression. These findings suggest that the efficacy of emotion regulation strategies in modulating valence and arousal may be contingent upon the individual’s AS, with distinct patterns emerging for Secure versus Fearful AS groups. Significance levels are denoted as follows: *p < .05, **p < .01, ***p < .001, ****p < .0001. The error bars represent standard deviations

In order to explore the nuances of the interactions and principal effects delineated by the two-way ANOVAs, the Sidak post-hoc test was utilized. In our exploration of valence, several notable results emerged. Specifically, a significant difference was observed between the Secure AS group under the reappraise strategy and the Anxious AS group under the suppress strategy (*d* = 0.979; mean difference = 0.8790; 95% CI [0.1002, 1.658], *p* = .0127). Similarly, the Secure AS group under the reappraise strategy differed significantly from the Fearful AS group under the suppress strategy (*d* = 0.917; mean difference = 0.8350; 95% CI [0.1011, 1.569], *p* = .0115) (Fig. 6A).

In the context of arousal, Sidak’s multiple comparisons yielded significant findings as well. The Secure AS group under the reappraise strategy demonstrated a significant decrease in arousal compared to the Fearful AS group under the reappraise strategy (mean difference = -1.109; 95% CI [-2.059, -0.1592], *p* = .0081). Additionally, the arousal level for the Secure AS group under reappraise was significantly lower compared to the Fearful AS group under the suppress strategy (mean difference = -1.407; 95% CI [-2.357, -0.4572], *p* = .0002). Another notable difference was between the Avoidant AS group under reappraise and the Fearful AS group under suppress (mean difference = -1.117 (95% CI [-2.160, -0.07450], *p* = .0238). Lastly, a marginal difference in arousal was observed between the Anxious AS group under reappraise and the Fearful AS group under suppress (mean difference = -1.158; 95% CI [-2.310, -0.006082], *p* = .0474) (Fig. 6B).

These results suggest nuanced interactions and distinct patterns in emotional responses, particularly in arousal, across different attachment styles when subjected to reappraisal and suppression strategies.

## Discussion

The results of this study provide valuable insights into the emotional regulation abilities of individuals with different attachment styles. Consistent with attachment theory, the analysis of the ECR-12 questionnaire revealed higher levels of anxiety attachment compared to avoidance attachment among the participants (Fig. 2A). This is in line with studies that have consistently shown these results in the general population, namely Chopik and Edelstein [65], Karataş et al. [66], and Guzmán-González et al. [48]. While these results may be influenced by cultural factors, there is a limited body of research assessing the distribution of attachment dimensions in Latin America. Cross-cultural studies examining attachment reveal that in societies with lower levels of individualism, anxiety within relationships tends to be more pronounced compared to Westernized countries, as demonstrated by Wang and Mallinckrodt [67]. This trend could potentially be applicable to the Latin American context, although more conclusive studies are necessary to support this claim. In relation to Chile, the findings obtained by Garrido et al. [68] are worth considering. They evaluated the distribution of adult attachment in Chile using the CaMir instrument and found that among insecure attachments, anxious (preoccupied) attachment was the most prevalent.

In terms of emotion regulation strategies, the current study employed cognitive reappraisal and expressive suppression. These strategies have been extensively studied in the field of emotion regulation. Cognitive reappraisal involves reinterpreting the meaning of a situation to alter emotional responses [6], while expressive suppression aims to inhibit the outward expression of emotions [17]. It is important to note that emotional response suppression, as a response-focused strategy, involves not only reducing the outward behavioral expression of negative emotions but also inadvertently diminishing the expression of positive emotions. While it may seem effective in controlling the external manifestations of negative feelings, suppression does not necessarily aid in lessening the internal experience of these emotions. In fact, this strategy might consume cognitive resources that would otherwise contribute to more effective social interactions and emotional management [7]. Proper emotional management typically entails acknowledging and accepting one’s emotions, fully experiencing them, and then potentially reassessing them [69, 70]. However, the habitual use of suppression can lead to a significant internal-external incongruence, where there is a discrepancy between what is felt internally and what is expressed outwardly. This incongruence can result in negative self-perceptions and a sense of alienation from both oneself and others, impacting overall well-being and the quality of interpersonal relationships [8]. The results of this study support previous findings regarding the effectiveness of cognitive reappraisal in reducing levels of displeasure [6, 8]. Furthermore, our results support the notion that suppression as an emotion regulation strategy may be less effective in reducing displeasure compared to cognitive reappraisal, especially in individuals with secure attachment styles [71, 72]. The limited effectiveness of suppression in reducing displeasure is consistent with studies emphasizing its potential negative consequences, such as increased physiological arousal and cognitive costs [8, 73].

All groups (secure, anxious, avoidant, and fearful) demonstrated the ability to effectively decrease displeasure through cognitive reappraisal compared to the ‘Natural-Negative’ condition (Figs. 3A and C and 4A, and 4C). These findings are consistent with studies highlighting the adaptive nature of cognitive reappraisal in regulating negative emotions across different populations [74–76]. Based on our findings, it is possible to infer that individuals with insecure attachment tendencies might effectively utilize reappraisal strategies to reduce displeasure if they receive appropriate instruction. However, individuals with anxious attachment exhibited difficulties in reducing arousal levels using any of the instructed strategies, which aligns with research highlighting the challenges of regulating arousal in individuals with high attachment anxiety [35, 77, 78].

In contrast, expressive suppression was found to be less effective in regulating emotions in the present study. The secure attachment style group, similar to previous research [37, 73], exhibited similar levels of displeasure when employing expressive suppression compared to passively viewing negative images (Fig. 3A). This suggests that individuals with secure attachment styles may use cognitive reappraisal more effectively rather than suppression to regulate their emotions effectively.

Furthermore, the findings for the avoidant attachment style group provide insights into the differential effectiveness of emotion regulation strategies. While both cognitive reappraisal and expressive suppression effectively reduced displeasure levels, attempts to suppress emotions resulted in similar levels of arousal compared to the ‘Natural-Negative’ condition (Fig. 4A and B). These results are in line with studies suggesting that individuals with avoidant attachment styles rely on suppression as a maladaptive emotion regulation strategy, with the cost of heightened physiological arousal consequently [35, 79, 80].

The fearful attachment style group demonstrated successful regulation of both displeasure and arousal using a cognitive reappraisal strategy (Fig. 4C and D). This finding may appear odd since Individuals with a fearful attachment style often exhibit higher levels of anxiety and fear [35]. This heightened emotionality can make it more challenging for them to regulate their emotions effectively, as they may experience intense and overwhelming emotional reactions. However, an alternative interpretation of these results could be considering studies that highlight the adaptive nature of emotion regulation in individuals with fearful attachment styles. These individuals often exhibit both approach and avoidance tendencies in regulating their emotions [35, 81]. This dual approach-avoidance pattern may allow them to effectively regulate their emotions by employing regulation strategies when they are instructed to do it in a more structured context, such as in our experiments. Another factor influencing this could be that these individuals often have heightened sensitivity to emotional cues and a strong motivation to manage their emotions due to their attachment-related insecurities [35]. This heightened awareness and motivation may contribute to their successful regulation of both displeasure and arousal in our structured experiment. Moreover, it is important to recognize that attachment styles and emotion regulation are complex and multifaceted constructs. Various factors, including individual differences, contextual factors, and the interplay between attachment and other psychological processes, can influence the regulation of emotions [35]. These factors may help explain the unexpected, yet adaptive regulation patterns observed in individuals with fearful attachment styles.

When directly comparing via intergroup analysis, the Secure AS group’s proficiency in utilizing cognitive reappraisal to effectively reduce arousal levels stands out, particularly when juxtaposed with the Fearful AS group. This underscores the potential of Secure AS individuals to leverage cognitive strategies to modulate physiological arousal effectively, which is consistent with their predisposition towards positive emotionality and resilience. In contrast, the lack of significant intergroup variations in valence during reappraisal might suggest a generalizable effect of this strategy across different attachment styles. Notwithstanding, the Secure AS and Avoidant AS groups exhibited higher valence scores when engaging in reappraisal, compared to the baseline ‘Natural-Negative’ condition. This finding may indicate a particular efficacy of reappraisal in reducing displeasure for these groups. The two-way ANOVA analysis further solidifies the independent effects of attachment styles and emotion regulation strategies on emotional outcomes, with no significant interaction observed. This could imply that the impact of attachment on emotion regulation is not contingent on the type of strategy employed.

The current study provides insights into the impact of attachment styles on the efficacy of emotion regulation strategies. However, one limitation that should be acknowledged is the sample size. Our sample was composed of 98 Chilean Latin-American adults. A larger sample size could potentially offer a more robust and generalizable understanding of the nuanced interactions between attachment styles and emotion regulation strategies. This is especially pertinent given the diverse nature of emotional experiences and the multifaceted constructs of attachment and emotion regulation. A bigger and more diverse sample might have provided a broader perspective, capturing a wider range of variability in attachment styles and emotional regulation capabilities across different populations. Furthermore, while our study makes significant contributions to the field, the results need to be interpreted with caution, considering the limitations posed by the sample size. Future research, ideally with larger and more diverse samples, is essential to validate and expand upon our findings, potentially offering even deeper insights into the complex dynamics of emotion regulation across different attachment styles.

In light of the potential concerns raised regarding the use of predefined cut-off values for categorizing attachment styles, we acknowledge the potential limitations of applying these thresholds without considering the unique characteristics of our sample. Although using large-sample-derived cut-off values to inform individual-level assessments is recommended [49], the reliance on cut-off values established from a different sample could introduce biases or inaccuracies in categorization. To mitigate these concerns a more sample-specific approach to determining cut-off values could be used in future research. One such approach could involve using the median scores within the current research sample to categorize attachment styles. Adopting a median-based categorization approach could also address variability in attachment expressions across different populations, ensuring that the categorization is both relevant and specific to the sample in question.

Another limitation of our study pertains to relying solely on self-report measures, which restrict our access to subjective experiences of the emotional process. The current methodology for assessing emotion regulation effectiveness primarily focuses on evaluating the influence of reappraisal on one’s personal experience rather than directly examining how suppression affects outward expression. To address this limitation, future research should aim to incorporate assessments of expressive outcomes of emotions and consider the physiological aspects that indicate successful or unsuccessful regulation. By expanding beyond self-reported emotional experiences, we can gain a more comprehensive understanding of the effectiveness of emotion regulation techniques. Another limitation is the potential variability in how participants interpret and apply the suppression and reappraisal strategies during the emotion regulation task. Even with clear instructions, individual differences in understanding and implementing these strategies can lead to heterogeneous approaches within the same condition. This variability can introduce noise into the data, making it challenging to draw definitive conclusions about the effectiveness of these strategies. For instance, one participant’s method of cognitive reappraisal could significantly differ from another’s, leading to variations in emotional outcomes that are not strictly due to their attachment style but rather to their unique approach to the strategy. To mitigate this, our study included structured and detailed guidelines for these strategies and the use of pre-task training to ensure a more uniform application of the strategies among participants. However, in line with this issue, our experimental design approached suppression and reappraisal as if they were mutually exclusive and could be distinctly invoked through instruction. However, in real-world scenarios, individuals rarely rely on a single strategy in isolation. Emotion regulation is a dynamic process where multiple strategies can be concurrently or sequentially employed, often blending together in a complex interplay that is influenced by context, individual differences, and the nature of the emotional stimulus itself [82]. Recognizing this, our study’s structured approach to eliciting specific emotion regulation strategies may not fully encapsulate the nuanced ways in which these strategies naturally occur and interact within individuals. While our instructions aimed to isolate the effects of suppression and reappraisal to examine their distinct mechanisms and outcomes, it is conceivable that participants might have inadvertently engaged in a combination of strategies despite the experimental conditions designed to elicit a particular response. This introduces an additional layer of variability, as the exclusive occurrence of one strategy over another is challenging to enforce and verify in a controlled environment. To address this complexity, we acknowledge that the binary classification of emotion regulation strategies in experimental settings does not fully reflect their fluid and overlapping nature in everyday emotion regulation [83]. Future research could benefit from incorporating methodologies that allow for the examination of these strategies in a more integrated manner, potentially through the use of real-time reporting or ecological momentary assessment techniques [83]. These approaches could provide deeper insights into how individuals naturally navigate between and combine different emotion regulation strategies in response to varying emotional challenges. In light of this, while our findings contribute valuable insights into the differential effects of suppression and reappraisal as instructed in a laboratory context, we caution against overgeneralizing these results to naturalistic emotion regulation processes. We advocate for further studies that explore the synergistic and context-dependent use of emotion regulation strategies to enrich our understanding of this complex psychological phenomenon.

One inherent limitation of conducting laboratory-based studies on emotional regulation, particularly those involving structured tasks preceded by training sessions, is the potential for such preparatory activities to introduce priming effects that could influence participants’ responses. This concern is especially pertinent in psychological experiments designed to investigate sensitive constructs, such as emotional regulation and attachment styles. Priming, in this context, refers to the subtle shaping of participants’ perceptions, attitudes, or behaviors as a result of exposure to specific stimuli or instructions before the main experimental tasks [84]. Given the complexity and sensitivity of the constructs under study, it is crucial to examine the extent to which pre-experimental training might affect the outcomes of such research. Moreover, another limitation was the reliance on laboratory-based assessments to gauge the effectiveness of emotion regulation strategies. While such controlled environments are beneficial for reducing extraneous variables and enhancing experimental rigor, they may not accurately reflect the complexities and nuances of real-world emotional experiences. Participants’ responses in a lab setting might not align with how they would naturally react in their everyday lives, where the context and stakes of emotional experiences can be vastly different. The ecological validity of the findings might be enhanced by incorporating naturalistic methods such as experience sampling or diary studies, where participants report on their emotion regulation efforts and outcomes in their daily lives. This approach can provide valuable information about how attachment styles influence emotion regulation in more typical, less controlled environments [37].

However, our adaptation of the emotion regulation paradigm holds promise for advancing our understanding in this area. By integrating our experimental design with advanced neuroimaging techniques such as functional Magnetic Resonance Imaging (fMRI) [85], Electroencephalogram (EEG) [59, 86–88], or eye-tracking systems [89–91], we can gain valuable insights into the dynamics of brain activity and pupil responses during emotional regulation. These additional measures can provide a more comprehensive picture of how attachment patterns interact with emotional regulation strategies and their associations with behavioral indicators of emotional experiences. This approach would allow us to go beyond subjective self-reporting and delve into objective measures to enhance the assessment of emotional regulation outcomes.

## Conclusions

These findings suggest that cognitive reappraisal was effective across all attachment styles in reducing displeasure, particularly among individuals with secure and avoidant attachment styles. Furthermore, the capacity of cognitive reappraisal to reduce arousal varies across attachment styles, with secure and fearful styles showing more effective regulation. In comparison, expressive suppression was less effective than reappraisal in reducing displeasure across most attachment styles and often associated with higher arousal levels, indicating it may be a less adaptive strategy for emotion regulation. Additionally, these results suggest that while emotion regulation strategies and attachment styles each independently influence emotional valence and arousal, their combined interaction does not significantly affect these emotional responses within our sample. Overall, these findings highlight the critical role of considering attachment styles when exploring individual differences in emotion regulation. Understanding the nuanced relationship between attachment styles and the efficacy of emotion regulation strategies can provide valuable insights into personalized approaches in psychological interventions and research.

### Electronic supplementary material

Below is the link to the electronic supplementary material.

Supplementary Material 1

## Data Availability

The data that support the findings of this study are available from the Universidad Católica del Norte (UCN). Data are available from the authors with the permission of the UCN and upon request.
