# Supplementary material for: Emotion regulation unveiled through the categorical lens of attachment
Source: BMC Psychol. 2024 Apr 27;12:240. doi: 10.1186/s40359-024-01748-z (PMC11056069; doi:10.1186/s40359-024-01748-z)
Supplement: Supplementary file 1 — Supplementary Material 1 [file 40359_2024_1748_MOESM1_ESM.docx]

1. **Supplementary Material**
   1. **Supplementary Tables**

**Table S1. Chosen IAPS Images for Each Experimental Condition (Lang et al., 2005)**

|  | **Experimental Conditions** | | | |
| --- | --- | --- | --- | --- |
| **Number (N°)** | **Natural-negative** | **Suppress** | **Reappraise** | **Natural-neutral** |
| 1 | 2683 | 2095 | 2130 | 7009 |
| 2 | 2703 | 2205 | 2700 | 7011 |
| 3 | 2710 | 2900 | 2751 | 7012 |
| 4 | 2717 | 6211 | 2811 | 7018 |
| 5 | 3220 | 6231 | 3550 | 7042 |
| 6 | 3230 | 6250 | 6260 | 7045 |
| 7 | 3500 | 6312 | 6350 | 7061 |
| 8 | 6300 | 6560 | 6530 | 2190 |
| 9 | 6315 | 6563 | 6561 | 2215 |
| 10 | 6360 | 6838 | 6562 | 2359 |
| 11 | 6540 | 9041 | 6825 | 2499 |
| 12 | 6550 | 9250 | 6834 | 2518 |
| 13 | 6840 | 9421 | 9050 | 2521 |
| 14 | 9429 | 9427 | 9419 | 2593 |
| 15 | 9900 | 9435 | 9425 | 2594 |

**Table S2. Descriptive Statistics for chosen IAPS Pictures across different conditions**

|  | **Natural-negative** | | **Suppress** | | **Reappraise** | | **Natural-neutral** | |
| --- | --- | --- | --- | --- | --- | --- | --- | --- |
| **Statistics** | **Mean** | **SD** | **Mean** | **SD** | **Mean** | **SD** | **Mean** | **SD** |
| **Valence** | 2.48 | 0.40 | 2.50 | 0.50 | 2.77 | 0.51 | 5.30 | 0.50 |
| **Arousal** | 6.09 | 0.59 | 5.76 | 0.81 | 5.83 | 0.81 | 3.48 | 0.48 |

**SD:** Standard Deviation.

**Table S3. Normality Test for ECR-12 scores: anxiety and avoidance dimensions**

| Shapiro-Wilk test | Anxiety ECR-12 | Avoidance ECR-12 |
| --- | --- | --- |
| W | 0.9748 | 0.9188 |
| P value | 0.0563 | <0.0001 |
| Passed normality test (alpha=0.05)? | Yes | No |
| P value summary | ns | **** |

**Table S4. Normality Test for valence during the emotion regulation task**

|  | Emotion regulation task conditions | | | |
| --- | --- | --- | --- | --- |
| Shapiro-Wilk test | Natural-Negative | Suppress | Reappraise | Natural-Neutral |
| W | 0.8621 | 0.9265 | 0.9530 | 0.8729 |
| P value | <0.0001 | <0.0001 | 0.0096 | <0.0001 |
| Passed normality test (alpha=0.05)? | No | No | No | No |
| P value summary | **** | **** | ** | **** |

**Table S5. Normality Test for arousal during the emotion regulation task**

|  | Emotion regulation task conditions | | | |
| --- | --- | --- | --- | --- |
| Shapiro-Wilk test | Natural-Negative | Suppress | Reappraise | Natural-Neutral |
| W | 0.9592 | 0.9252 | 0.9173 | 0.8727 |
| P value | 0.0040 | <0.0001 | <0.0001 | <0.0001 |
| Passed normality test (alpha=0.05)? | No | No | No | No |
| P value summary | ** | **** | **** | **** |

**Table S6. Normality Test for valence during the emotion regulation task: Secure Attachment Style**

|  | Emotion regulation task conditions | | | |
| --- | --- | --- | --- | --- |
| Shapiro-Wilk test | Natural-Negative | Suppress | Reappraise | Natural-Neutral |
| W | 0.9592 | 0.8814 | 0.8939 | 0.7672 |
| P value | <0.0001 | <0.0001 | 0.0015 | <0.0001 |
| Passed normality test (alpha=0.05)? | No | No | No | No |
| P value summary | **** | **** | ** | **** |

**Table S7. Normality Test for arousal during the emotion regulation task: Secure Attachment Style**

|  | Emotion regulation task conditions | | | |
| --- | --- | --- | --- | --- |
| Shapiro-Wilk test | Natural-Negative | Suppress | Reappraise | Natural-Neutral |
| W | 0.9434 | 0.9109 | 0.9084 | 0.8701 |
| P value | 0.0493 | 0.0046 | 0.0039 | 0.0003 |
| Passed normality test (alpha=0.05)? | -- | No | No | No |
| P value summary | ns | ** | ** | **** |

**Table S8. Normality Test for valence during the emotion regulation task: Anxious Attachment Style**

|  | Emotion regulation task conditions | | | |
| --- | --- | --- | --- | --- |
| Shapiro-Wilk test | Natural-Negative | Suppress | Reappraise | Natural-Neutral |
| W | 0.9636 | 0.9622 | 0.9458 | 0.8622 |
| P value | 0.7279 | 0.7014 | 0.4264 | 0.0207 |
| Passed normality test (alpha=0.05)? | Yes | Yes | Yes | No |
| P value summary | ns | ns | ns | ** |

**Table S9. Normality Test for arousal during the emotion regulation task: Anxious Attachment Style**

|  | Emotion regulation task conditions | | | |
| --- | --- | --- | --- | --- |
| Shapiro-Wilk test | Natural-Negative | Suppress | Reappraise | Natural-Neutral |
| W | 0.9641 | 0.8919 | 0.9227 | 0.8309 |
| P value | 0.7356 | 0.0597 | 0.1866 | 0.0072 |
| Passed normality test (alpha=0.05)? | Yes | Yes | Yes | No |
| P value summary | ns | ns | ns | ** |

**Table S10. Normality Test for valence during the emotion regulation task: Avoidant Attachment Style**

|  | Emotion regulation task conditions | | | |
| --- | --- | --- | --- | --- |
| Shapiro-Wilk test | Natural-Negative | Suppress | Reappraise | Natural-Neutral |
| W | 0.9599 | 0.9455 | 0.9302 | 0.9533 |
| P value | 0.4362 | 0.2158 | 0.0985 | 0.3192 |
| Passed normality test (alpha=0.05)? | Yes | Yes | Yes | Yes |
| P value summary | ns | ns | ns | ns |

**Table S11. Normality Test for arousal during the emotion regulation task: Avoidant Attachment Style**

|  | Emotion regulation task conditions | | | |
| --- | --- | --- | --- | --- |
| Shapiro-Wilk test | Natural-Negative | Suppress | Reappraise | Natural-Neutral |
| W | 0.9165 | 0.8504 | 0.8912 | 0.9145 |
| P value | 0.0490 | 0.0022 | 0.0141 | 0.0441 |
| Passed normality test (alpha=0.05)? | -- | No | No | No |
| P value summary | ns | ** | * | ** |

**Table S12. Normality Test for valence during the emotion regulation task: Fearful Attachment Style**

|  | Emotion regulation task conditions | | | |
| --- | --- | --- | --- | --- |
| Shapiro-Wilk test | Natural-Negative | Suppress | Reappraise | Natural-Neutral |
| W | 0.9490 | 0.9673 | 0.9550 | 0.8845 |
| P value | 0.3796 | 0.7216 | 0.4785 | 0.0257 |
| Passed normality test (alpha=0.05)? | Yes | Yes | Yes | No |
| P value summary | ns | ns | ns | N* |

**Table S13. Normality Test for arousal during the emotion regulation task: Fearful Attachment Style**

|  | Emotion regulation task conditions | | | |
| --- | --- | --- | --- | --- |
| Shapiro-Wilk test | Natural-Negative | Suppress | Reappraise | Natural-Neutral |
| W | 0.9727 | 0.9757 | 0.9554 | 0.8826 |
| P value | 0.8288 | 0.8811 | 0.4862 | 0.0238 |
| Passed normality test (alpha=0.05)? | Yes | Yes | Yes | No |
| P value summary | ns | ns | ns | N* |

**Table S14. Dunn Test Comparisons of Valence Values During 'Reappraise' Across Different Attachment Styles.**

| **Comparisons** | **Cohen's d** | **Mean rank diff** | **95% CI** | **p-value** |
| --- | --- | --- | --- | --- |
| Secure AS vs. Anxious AS | 0.588 | 14.73 | 0.095 to 1.081 | 0.4845 |
| Secure AS vs. Avoidant AS | 0.121 | -3.301 | -0.354 to 0.596 | >0.9999 |
| Secure AS vs. Fearful AS | 0.621 | 14.71 | 0.134 to 1.107 | 0.3851 |
| Anxious AS vs. Avoidant AS | -0.599 | -18.03 | -1.217 to 0.018 | 0.2955 |
| Anxious AS vs. Fearful AS | 0.041 | -0.01809 | -0.621 to 0.703 | >0.9999 |
| Avoidant AS vs. Fearful AS | 0.628 | 18.01 | 0.031 to 1.226 | 0.2337 |

**Table S15. Dunn Test Comparisons of Arousal Values During 'Reappraise' Across Different Attachment Styles.**

| **Comparisons** | **Cohen's d** | **Mean rank diff** | **95% CI** | **p-value** |
| --- | --- | --- | --- | --- |
| Secure AS vs. Anxious AS | -0.272 | -7.780 | -0.872 to 0.327 | >0.9999 |
| Secure AS vs. Avoidant AS | -0.302 | -7.968 | -0.831 to 0.227 | >0.9999 |
| Secure AS vs. Fearful AS | -1.080 | -28.35 | -1.696 to -0.464 | 0.0022 |
| Anxious AS vs. Avoidant AS | -0.040 | -0.1875 | -0.660 to 0.580 | >0.9999 |
| Anxious AS vs. Fearful AS | -0.759 | -20.57 | -1.409 to -0.109 | 0.1978 |
| Avoidant AS vs. Fearful AS | -0.712 | -20.38 | -1.326 to -0.097 | 0.1173 |

**Table S16. Dunn Test Comparisons of Valence Values During 'Reappraise' Among Different Attachment Styles and the Natural-Negative Condition.**

| **Comparisons** | **Cohen's d** | **Mean rank diff** | **95% CI** | **p-value** |
| --- | --- | --- | --- | --- |
| Natural-Negative vs. Secure AS | 0.942 | -54.78 | -0.002 to 0.813 | <0.0001 |
| Natural-Negative vs. Anxious AS | 0.405 | -30.19 | -0.660 to 0.580 | 0.1931 |
| Natural-Negative vs. Avoidant AS | 0.871 | -59.82 | 0.487 to 1.255 | <0.0001 |
| Natural-Negative vs. Fearful AS | 0.375 | -28.42 | -0.023 to 0.774 | 0.1823 |

**Table S17. Dunn Test Comparisons of Arousal Values During 'Reappraise' Among Different Attachment Styles and the Natural-Negative Condition.**

| **Comparisons** | **Cohen's d** | **Mean rank diff** | **95% CI** | **p-value** |
| --- | --- | --- | --- | --- |
| Natural-Negative vs. Secure AS | -0.737 | 42.53 | -1.060 to -0.413 | 0.0003 |
| Natural-Negative vs. Anxious AS | -0.498 | 28.25 | -0.938 to -0.059 | 0.2587 |
| Natural-Negative vs. Avoidant AS | -0.464 | 27.38 | -0.869 to -0.059 | 0.1362 |
| Natural-Negative vs. Fearful AS | 0.207 | -12.83 | -0.289 to 0.703 | >0.9999 |

**Table S18. Dunn Test Comparisons of Valence Values During 'Suppress' Across Different Attachment Styles.**

| **Comparisons** | **Cohen's d** | **Mean rank diff** | **95% CI** | **p-value** |
| --- | --- | --- | --- | --- |
| Secure AS vs. Anxious AS | 0.650 | 18.41 | 0.179 to 1.121 | 0.1747 |
| Secure AS vs. Avoidant AS | 0.338 | 6.740 | -0.135 to 0.811 | >0.9999 |
| Secure AS vs. Fearful AS | 0.592 | 16.12 | 0.106 to 1.079 | 0.2552 |
| Anxious AS vs. Avoidant AS | -0.410 | -11.67 | -1.013 to 0.194 | >0.9999 |
| Anxious AS vs. Fearful AS | -0.068 | -2.283 | -0.722 to 0.586 | >0.9999 |
| Avoidant AS vs. Fearful AS | 0.325 | 9.384 | -0.274 to 0.923 | >0.9999 |

**Table S19. Dunn Test Comparisons of Arousal Values During 'Suppress' Across Different Attachment Styles.**

| **Comparisons** | **Cohen's d** | **Mean rank diff** | **95% CI** | **p-value** |
| --- | --- | --- | --- | --- |
| Secure AS vs. Anxious AS | -0.283 | -7.781 | -0.872, 0.305 | >0.9999 |
| Secure AS vs. Avoidant AS | -0.402 | -10.85 | -0.925, 0.122 | 0.8462 |
| Secure AS vs. Fearful AS | -1.109 | -28.74 | -1.705, -0.514 | 0.0018 |
| Anxious AS vs. Avoidant AS | -0.125 | -3.073 | -0.746, 0.495 | >0.9999 |
| Anxious AS vs. Fearful AS | -0.797 | -20.96 | 1.450, -0.144 | 0.1787 |
| Avoidant AS vs. Fearful AS | -0.664 | -17.88 | -1.275, -0.054 | 0.2428 |

**Table S20. Dunn Test Comparisons of Valence Values During 'Suppress' Among Different Attachment Styles and the Natural-Negative Condition.**

| **Comparisons** | **Cohen's d** | **Mean rank diff** | **95% CI** | **p-value** |
| --- | --- | --- | --- | --- |
| Natural-Negative vs. Secure AS | 0.636 | -39.75 | 0.258 to 1.013 | 0.0009 |
| Natural-Negative vs. Anxious AS | 0.034 | -5.967 | -0.340 to 0.408 | >0.9999 |
| Natural-Negative vs. Avoidant AS | 0.336 | -26.78 | -0.044 to 0.717 | 0.1523 |
| Natural-Negative vs. Fearful AS | 0.082 | -9.460 | -0.317 to 0.480 | >0.9999 |

**Table S21. Dunn Test Comparisons of Arousal Values During 'Suppress' Among Different Attachment Styles and the Natural-Negative Condition.**

| **Comparisons** | **Cohen's d** | **Mean rank diff** | **95% CI** | **p-value** |
| --- | --- | --- | --- | --- |
| Natural-Negative vs. Secure AS | -0.366 | 24.16 | -0.749 to 0.017 | 0.0976 |
| Natural-Negative vs. Anxious AS | -0.066 | 8.777 | -0.634 to 0.502 | >0.9999 |
| Natural-Negative vs. Avoidant AS | 0.076 | 3.402 | -0.426 to 0.578 | >0.9999 |
| Natural-Negative vs. Fearful AS | 0.874 | -38.44 | 0.269 to 1.479 | 0.0273 |
